# Structures of DnaA domain I reveal a dimer conserved across Actinomycetes

**DOI:** 10.1093/nar/gkag596

**Published:** 2026-06-16

**Authors:** Porter K Ellis, Bindu Y Srinivasu, Jose Chavez Orozco, Gregory A Wray, Thomas E Wales, Maria A Schumacher

**Affiliations:** Department of Biochemistry, 307 Research Dr., Box 3711, Duke University Medical Center, Durham, NC 27710, United States; Department of Chemistry and Chemical Biology, Northeastern University, Boston, MA 02115, United States; Department of Biochemistry, 307 Research Dr., Box 3711, Duke University Medical Center, Durham, NC 27710, United States; Department of Biology, 130 Science Dr., Duke University, Durham, NC 27708, United States; Department of Chemistry and Chemical Biology, Northeastern University, Boston, MA 02115, United States; Department of Biochemistry, 307 Research Dr., Box 3711, Duke University Medical Center, Durham, NC 27710, United States

## Abstract

DNA replication is a fundamental process in biology, and initiation marks a key regulatory step. In bacteria, DNA replication is initiated by the DnaA protein. DnaA exhibits multidomain architecture, consisting of an N-terminal domain I, linker region, AAA+ family ATPase cassette, and C-terminal DNA-binding motif. Taxon-specific regulatory functions are primarily coordinated by the DnaA domain I (DnaA^DI^), which exhibits substantial sequence variation across bacteria. Notably, although DnaA^DI^ has been shown to be essential, its contributions to initiation are not completely understood. Previous studies suggested a role for DnaA^DI^ in the assembly of the initiation complex at the origin. However, the molecular mechanisms behind DnaA^DI^ functions have not been resolved. Here, we report the DnaA^DI^ structures from 10 species in the class Actinomycetes. Strikingly, all structures reveal the same, unique dimer, and our analyses show that key elements that support DnaA^DI^ self-interaction are broadly conserved across the class Actinomycetes. Further, a suite of biochemical oligomerization assays and HDX-MS (hydrogen–deuterium exchange mass spectrometry) studies support the formation of dimers with µM affinities. These findings suggest that weak DnaA^DI^ dimerization, which is a broadly conserved mechanism across the Actinomycetes, likely contributes to proper replication initiation in these bacteria.

## Graphical Abstract

**Graphical Abstract:**
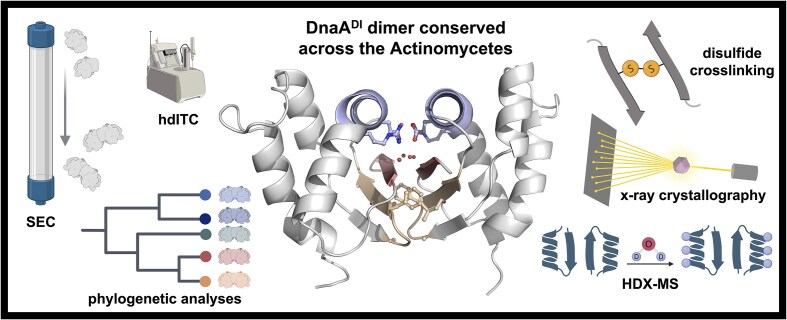

## Introduction

DNA replication is among the most fundamental of biological processes, essential to the faithful transmission of genetic information from mother to daughter cell. In all life, DNA replication proceeds through three essential stages: initiation, elongation, and termination [1]. In bacteria, during initiation, duplex DNA is unwound at a specific chromosomal site, called the origin of chromosomal replication (*oriC)*, to facilitate loading of the replisome machinery and thereby establish the nascent replication fork [2]. Initiation events are exquisitely regulated, reflecting the severe consequences of untimely chromosome synthesis [3, 4]. In bacteria, the essential and conserved protein DnaA, called the initiator, is responsible for executing replication initiation and is the central object of diverse, taxon-specific DNA-replication regulation mechanisms [5, 6].

**Figure 1.**
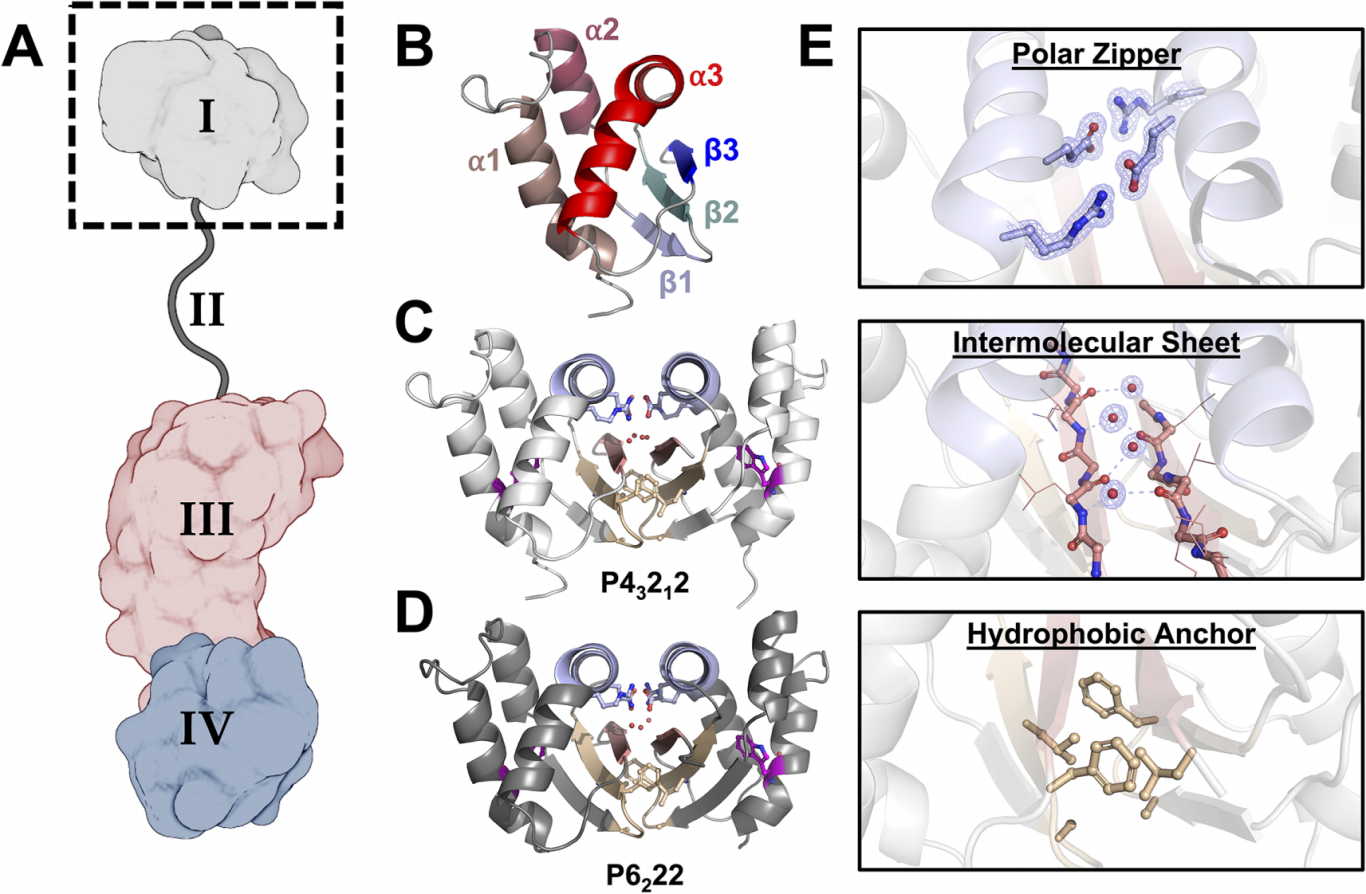
DnaA^DI^ domain organization and structures of the *Rhodococcus hoagii* (*Rh*) DnaA^DI^. (**A**) Schematic overview of the DnaA modular domain architecture. The C-terminal domains III and IV are shown in light pink and blue, respectively. The N-terminal domains I and II are shown in gray. DnaA^DI^, the object of this study, is indicated with a box. This panel was created in BioRender. Ellis, P. (2026) https://BioRender.com/gu7xjc5j. (**B**) The *Rh* DnaA^DI^ ⍺1-⍺2-β1-β2-⍺3-β3 secondary structure diagram. Helices and strands are colored in shades of red and blue, respectively. (**C**) The *Rh* DnaA^DI^ dimer structure in the P4_3_2_1_2 spacegroup. The polar zipper, intermolecular β-sheet, and hydrophobic anchor dimerization elements are depicted in light blue, salmon, and wheat, respectively. Residues involved in select intermolecular contacts are shown in ball-and-stick representation. W12, equivalent to W6 thought to support dimerization in the *E. coli* DnaA^DI^, is depicted in magenta. (**D**) The *Rh* DnaA^DI^ dimer structure in the P6_2_22 spacegroup. Dimerization elements and W12 are depicted as described in panel (C). (**E**) Close up of *Rh* DnaA^DI^ dimerization elements with 2*m*F_O_ – DF_C_ electron density (blue mesh), contoured at 2.0 σ and a 1.6 Å carve radius included around select features of interest.

Much of our early understanding of DnaA activity and regulation comes from studies in *Escherichia coli* [5, 7–14]. Subsequent investigations in *Streptomyces, Mycobacterium, Caulobacter, Helicobacter* and other bacteria revealed that key DnaA functions are conserved across bacterial genera [15–23]. Critically, initiation in all bacteria proceeds through the orisome, a large, ordered assembly of DnaA molecules at *oriC*. Orisome formation is genetically determined, directed by the location and distribution of DnaA recognition sites, called DnaA boxes, which vary in affinity for DnaA [24]. Cooperative binding is a key feature of DnaA-box binding and the stepwise orisome assembly process [16, 21, 25, 26]. The functional consequence of orisome formation in all species is the unwinding of DNA and recruitment of the replisome. However, the specific architecture of the orisome is not conserved across bacterial phyla, suggesting that orisome remodeling represents an evolutionary strategy for regulating initiation events [27]. It has been proposed that diversity in orisome architecture is underpinned by a commensurate diversity of bacterial lifestyle [28]. The modular makeup of DnaA lends credence to this thesis (Fig. 1A): the C-terminal region of the protein encodes the highly conserved domains III and IV, which consist of AAA+ (ATPases associated with diverse cellular activities) ATPase and DNA-binding modules, respectively; the N-terminal region encodes the poorly conserved domains I and II. These latter domains have been shown to be responsible for taxon-specific regulatory functions and putative long-range oligomeric interactions [11, 29, 30]. Thus, the C-terminal region may be considered to encode basic DNA-recognition and orisome-scaffolding machinery, while the N-terminal region can be thought to encode an adaptable handle, fine-tuned to support the specific regulatory and architectural requirements of an organism’s orisome assembly process.

Domain II of DnaA encodes a flexible region and demonstrates the greatest variability across bacterial phyla. This region, ranging in length from only a few residues to over 200 residues, appears to promote the correct spatial arrangement of DnaA molecules within the orisome complex [30]. Domain I (herein called DnaA^DI^), unlike domain II, encompasses a folded domain of ∼100 residues. Importantly, data have shown that DnaA^DI^ is essential for the initiation function of DnaA. Although found to be dispensable for *oriC* unwinding *in vitro*, DnaA^DI^-deletion mutants are defective in DNA replication *in vitro*, and overexpression of DnaA^DI^ blocks initiation *in vivo* [13, 31, 32].

Data indicate that DnaA^DI^ functions as a protein–protein interaction hub, orchestrating the complex assembly of the orisome through interactions with replisome machinery and regulatory factors. Among these, *in vitro* experiments show that DnaA^DI^ directly binds the replicative helicase, DnaB [29, 33, 34]. Consistent with this, *in vivo* studies demonstrate that DnaA^DI^ is required for loading DnaB onto DNA [31]. Further, DnaA^DI^ has been shown to directly interact with taxon-specific regulatory proteins, including DiaA in *E. coli*, HobA in *Helicobacter pylori*, and SirA in *Bacillus subtilis*, which appear to stimulate or suppress DnaA oligomerization [35–38]. *E. coli* DnaA^DI^ has also been shown to bind the proteins Dps, Hda, HU, L2, and YfdR, which all appear to regulate replication initiation [39–43].

Additional data have indicated that DnaA^DI^ may form oligomers. These self-interactions are proposed to support cooperative binding of DnaA at *oriC* and mediate long-distance interactions between DnaA molecules bound to distally located DnaA boxes [13, 16, 29, 35, 37, 44, 45]. Indeed, biochemical and genetic experiments suggesting that DnaA^DI^ could form oligomers were first documented in *E. coli* and *Streptomyces lividans* over two decades ago [13, 15, 16, 29, 46]. In particular, *S. lividans* DnaA^DI^ self-interactions were detected using one-hybrid analyses, and it was proposed that such interactions may support orisome assembly [15, 16]. An NMR chemical-shift-perturbation study performed on *E. coli* DnaA^DI^ mapped a putative dimerization interface to helix ⍺1 and β1 with possible contributions from β2 and the N-terminus of β3 of the domain, but the study did not resolve the putative dimer structure [29, 46]. Other DnaA^DI^s, such as the *Mycoplasma genitalium* DnaA^DI^, may not form oligomers [47]. No further structural evidence is available to illuminate the mechanism of DnaA^DI^ oligomerization.

To expand our understanding of DnaA^DI^ structure and function across bacterial genera, we initiated studies on the *Mycobacterium tuberculosis* (*Mtb*) DnaA^DI^. The clinical relevance of mycobacteria and dearth of molecular and biological information on DnaA^DI^ in these species rendered it an interesting candidate for interrogation [20, 48]. Through our studies, we solved two different crystal structures of DnaA^DI^ from the related *Rhodococcus hoagii *(*Rh*). These structures revealed the same, unexpected dimer that is markedly distinct from that proposed for the *E. coli* DnaA^DI^ dimer. Remarkably, residues that support the *Rhodococcus* DnaA^DI^ dimer are conserved across other families of the order Mycobacteriales and, more broadly, the class Actinomycetes [49], suggesting the dimer may have a conserved physiological role. Concordant with this hypothesis, we solved DnaA^DI^ structures of nine additional organisms, representing four biological orders in the class Actinomycetes, all of which revealed the same dimer. Phylogenetic analyses and a battery of biochemical assays provide further evidence for the widespread conservation of this dimerization interface across the Actinomycetes. Accordingly, these results suggest an important function for DnaA^DI^ dimerization in the Actinomycetes DNA-replication program.

## Materials and methods

### Protein expression and purification

The sequences of all plasmids and recombinant proteins used in this study are provided in Supplementary Table S1. Briefly, codon-optimized (for *E. coli* expression) genes encoding DnaA^DI^ (WT and mutants) were purchased from GenScript Corporation and subcloned into the pET15b vector (using the NdeI and BamHI restriction sites) for expression of proteins encoding an N-terminal-histidine tag and thrombin cleavage site. Chemically competent C41(DE3) *E. coli* cells (Fisher Scientific) were transformed with expression plasmids by standard heat shock transformation methods. Cells harboring the DnaA^DI^ expression vector were grown at 37°C with shaking in Luria broth media (4.5–9.0 l) supplemented with 100 µg/ml ampicillin to an *A*_600_ of ∼0.4–0.6. Cells were then induced with 1 mM isopropyl-β-D-thiogalatopyranoside (IPTG) and left at 15°C with shaking overnight. In a typical protein preparation, cells were harvested by centrifugation at 4000 rpm at 4°C for 15 min and subsequently frozen at −20°C for temporary storage or immediately used for protein purification. No differences were observed between proteins prepared from fresh or frozen cell pellets. Cell suspensions were prepared to a final volume of 100 ml by resuspending pelleted *E. coli* (∼30 g wet mass from 4.5 l cultures) in Buffer A300 [25 mM Tris, pH 7.5; 300 mM NaCl; 5% (v/v) glycerol; 0.5 mM β-mercaptoethanol] supplemented with protease inhibitors (125 µM aprotinin, 1.25 µM leupeptin, 1.25 µM pepstatin) and DNase (0.01 mg/ml). The cell slurry was homogenized with a Dounce tissue grinder and subsequently sonicated to achieve cell lysis. The lysate was clarified by centrifugation at 16 000–19 000 rpm at 4°C for 1–2 h after which the soluble fraction was loaded onto a cobalt-charged IMAC (immobilized-metal affinity chromatography) column (TALON, TakaraBio). After lysate loading, the column was washed with Buffer A300 supplemented with 5–20 mM imidazole to remove contaminating proteins. DnaA^DI^ proteins were eluted from the column with 15 ml Buffer A300 fractions supplemented with imidazole in stepwise-increasing concentration. Fractions were analyzed by sodium dodecyl sulfate–polyacrylamide gel electrophoresis (SDS–PAGE) and assessed for purity. Pure fractions were pooled and concentrated using 3K cutoff centrifugal filters (Millipore) for subsequent analysis or further purification by size exclusion chromatography (SEC) using a HiLoad 26/600 Superdex 75pg column. DnaA^DI^ protein concentrations were determined using *A*_280_ values (NanoDrop) and theoretical extinction coefficients from Expasy ProtParam (https://web.expasy.org/protparam/). Note: For all but *Corynebacterium bovis* DnaA^DI^ (S89C), extinction coefficients were computed assuming all Cys residues are reduced; for *C. bovis* DnaA^DI^ (S89C), the extinction coefficient was computed assuming the Cys residue forms a disulfide bond (calculated by dividing the extinction coefficient of a tandem construct by two).

Purification of the *C. bovis* DnaA^DI^ (S89C) protein was carried out as above in Buffer A300 lacking reducing agent. Following IMAC, the DnaA^DI^ (S89C) mutant was purified predominantly in reduced form. Intermolecular disulfide bond formation was achieved by air oxidation catalyzed by CuSO_4_. Specifically, IMAC fractions were concentrated to ∼40 ml and added to a 50-ml Erlenmeyer flask. CuSO_4_ was added to the protein solution to a final concentration of 5–50 µM (within this concentration range, there was no notable difference in oxidation rate or protein quality). Upon addition of the Cu^2+^ catalyst, the protein solution immediately turned purple. The reaction was left at room temperature (rt) with stirring for 2–3 h to facilitate disulfide bond formation (after this time, the solution was colorless, and the reaction was assumed to be complete). The reaction was quenched with 1 mM ethylenediaminetetraacetic acid, pH 8.0. The oxidized protein was exchanged into Buffer A150 and further purified by SEC using a HiLoad 26/600 Superdex 75pg column. Disulfide-linked dimer formation was confirmed by tandem mass spectrometry (MS) and SDS–PAGE analysis of samples boiled in loading buffer with and without reducing agent.

### Crystallization and structure determination of DnaA^DI^ proteins

Protein concentrations (intrinsic absorbance), crystallization and cryogenic-preservation conditions, and data collection sources for each crystal are provided in Supplementary Table S2. For all DnaA^DI^ proteins (except for *Rh* DnaA^DI^, which crystallized in the presence of its purification tag), removal of the poly-histidine purification tag was carried out prior to crystallization trials. Tag removal was achieved using the Thrombin Cleavage Capture Kit (Millipore) according to manufacturer’s guidelines. Cleaved poly-histidine tags were captured by flowing the cleavage solution over a cobalt-charged IMAC column to liberate cleaved DnaA^DI^ proteins (note: each cleaved construct contained a short, N-terminal GSH tri-peptide that remains after cleavage in the thrombin reaction). Proteins were crystallized at rt using the vapor diffusion hanging drop method. Crystallization trials were performed using the Wizard Classic I-IV, MembFac, SaltRx1, and SaltRx2 screens, and initial conditions producing crystals were subsequently optimized for growth of large, single crystals. After identification of an appropriate cryo-preservation condition, crystals were preserved by plunging into a liquid nitrogen (LN2) bath or placing under an LN2 stream.

X-ray intensity data were collected at the Advanced Light Source on beamlines 5.0.1 and 5.0.2 or on a homesource XtaLab Synergy diffractometer equipped with a Cu PhotoJet X-ray generator, HyPix-6000HE detector, and 800 Series Cryostream Cooler. Data scaling was performed using XDS (for intensity data collected at ALS) or CrysAlis^Pro^ (for intensity data collected on the homesource) [50, 51]. X-ray intensity data were merged using Aimless as implemented in the CCP4 suite [52, 53]. Crystal structures were solved by molecular replacement as implemented in Phenix Phaser with experimental or AlphaFold3 single-subunit search models [54–56]. Model building was performed in Coot [57] and refinement, carried out to convergence, was performed using Phenix Refine [58]. Biomolecular representations were generated using PyMOL [59]. Intermolecular sheet (IMS) β3–β3′ interaction angles were computed using the *cafit_orientation* method as implemented in the AnglesBetweenHelices PyMOL function (script available at https://pymolwiki.org/AngleBetweenHelices; author: Thomas Holder). β3 and β3′ assignments for AnglesBetweenHelices analysis were as follows: *Rh* (tetragonal) residues 85–89*; Rhodococcus ruber* residues 82–87*; Nocardia asteroides* (trigonal) residues 87–91*; N. pseudobrasiliensis* residues 87–92*; Corynebacterium diphtheriae* residues 83–87*; S. lividans* (hexagonal) residues 83–88*; Bifidobacterium bifidum* residues 84–88; and *B. primatium* residues 81–86. X-ray merging and refinement statistics are found in Supplementary Table S3.

### Isothermal titration calorimetry

For isothermal titration calorimetry (ITC), purified DnaA^DI^ proteins were exchanged into Buffer A150 [25 mM Tris, pH 7.5; 150 mM NaCl; 5% (v/v) glycerol; 0.5 mM β-mercaptoethanol] and concentrated to ∼0.6–1.4 mM (effective monomer concentration). Stepwise titration of DnaA^DI^ into Buffer A150 was performed using a VP-ITC microcalorimeter with a 24- or 32-point injection schedule. Experiments were carried out at 25°C under atmospheric pressure with 199 rpm stirring. Data were analyzed in Origin 7 SR4 (v7.0552) and fit by non-linear regression using the “Dissociation” model. Automatic baseline detection and minor manual baseline corrections were used to define isotherms. The first (small) injection of each titration series was omitted from analysis. Each reported dissociation constant (K_D_) is given by the average of at least two replicates with error representing the standard deviation of the replicates (*n* = 3 for *S. lividans* DnaA^DI^; *n* = 2 for all remaining examined constructs). *Corynebacterium bovis* DnaA^DI^ (S89C) ITC experiments were carried out as above in Buffer A150 lacking reducing agent. Replicate experiments are provided in Supplementary Fig. S1.

### Size exclusion chromatography

*C. bovis, Rh, Mtb, E. coli*, and *H. pylori* DnaA^DI^ constructs were purified as above and subsequently exchanged into Buffer A150 (buffer exchange was accomplished using Millipore centricons, gel filtration, or dilution with Buffer A lacking NaCl). The *C. bovis, E. coli*, and *H. pylori* DnaA^DI^ proteins were concentrated to 2.0 mg/ml; the *Mtb* and *Rh* DnaA^DI^ proteins were concentrated to 0.75 mg/ml. DnaA^DI^ samples were injected over a HiLoad 26/600 Superdex 75pg column equilibrated in Buffer A150 and eluted from the column with a 320-ml isocratic elution in Buffer A150. Protein elution was monitored by intrinsic UV-absorbance. Molecular weight determination of the *C. bovis* DnaA^DI^ constructs was determined using a gel phase distribution coefficient calibration curve [K_av_ = (V_e_ - V_0_)/(V_C_ – V_0_)]. The void volume, V_0_, was determined using Blue Dextran (∼2000 kDa) and the column’s accessible volume, V_C_, is given by 320 ml. Elution volumes, V_e_, were determined for four protein standards: aprotinin, ∼6.5 kDa; cytochrome c, ∼12.4 kDa; carbonic anhydrase, 29 kDa; and bovine serum albumin, 66 kDa. Note: the *C. bovis* DnaA^DI^ (S89C) SEC experiments were performed in Buffer A150 lacking reducing agent.

### Circular dichroism

Circular dichroism (CD) spectra were collected for the *Rh* DnaA^DI^ wild type (WT) and mutant proteins using an AVIV CD-435 spectrophotometer. Experiments were carried out at 17.5 µM protein concentration in 25 mM KH_2_PO_4_/K_2_HPO_4_, 150 mM NaF, 5% (v/v) glycerol, and 0.5 mM β-mercaptoethanol at pH 7.5. For all samples and corresponding buffer blanks, spectra were collected in quintuplicate from 260–200 nm with 1-nm sampling at 25°C in a 1-mm quartz cuvette. Raw ellipticity was calculated as the average of quintuplicate readings. Baseline correction was performed by subtracting blank contributions to CD signal. Per-residue and protein concentration normalization were performed by dividing raw CD signal by the product of the assay protein concentration and protein sequence length. Mean residue ellipticity was plotted as a function of wavelength.

### Liquid chromatography-tandem mass spectrometry

For MS analysis, a *C. bovis* DnaA^DI^ (S89C) gel slice was destained by washing twice in 200 μl 40% (v/v) acetonitrile/60% (v/v) 50 mM NH_4_HCO_3_, pH 8, for 30 min on a rotator. The gel slice was then dehydrated briefly in 200 μl 100% (v/v) acetonitrile, before allowing to air dry at rt. The in-gel digestion was initiated by adding 30 μl of 0.01 μg/μl sequencing grade modified trypsin (Promega), which had been dissolved in 50 mM NH_4_HCO_3_, and an additional 30 μl of 50 mM NH_4_HCO_3_ and incubated at 37°C for 16 h with shaking. Following digestion, 60 μl extraction solution [1% TFA/2% acetonitrile, (v/v)] was added and the gel slices were incubated at rt for 30 min with occasional vortexing. Supernatant containing peptides was removed and an additional shrinking step in 50 μl neat acetonitrile was performed to enhance recovery. The two eluates were then combined and lyophilized to dryness overnight. The lyophilized sample was resuspended in 200 μl of 1% TFA/2% acetonitrile (v/v) prior to liquid chromatography-tandem mass spectrometry (LC-MS/MS) analysis.

Five percent of the gel band sample was subjected to LC-MS/MS using an EvoSep One UPLC coupled to a Thermo Orbitrap Astral high resolution accurate mass tandem mass spectrometer (Thermo). Briefly, 10 μl sample was loaded on an EvoTip (EvoSep) and eluted onto a 1.5-µm EvoSep 150 μm ID x 8 cm performance (EvoSep) column using the SPD60 gradient at 55°C. Data collection on the Orbitrap Astral mass spectrometer was performed in a data-dependent acquisition (DDA) mode of acquisition with an *r* = 120 000 (at *m*/*z* 200) full MS scan from *m*/*z* 350–1500 in the OT with a target AGC value of 4e5 ions. DDA scans with 1.2 *m*/*z* isolation were selected based on intensity and subjected to HCD at 28% with accumulation time of 10 ms in the Astral analyzer.

Raw LC-MS/MS data files were processed in Proteome Discoverer 3.1 (Thermo Scientific) and then submitted to independent Sequest database search against a *C. bovis* protein database containing both forward (5811 entries) and reverse entries of each protein, along with a custom sequence for the protein of interest. Search tolerances were 5 ppm for precursor ions and 0.02 Da for product ions using trypsin specificity with up to two missed cleavages. Crosslinks were identified using the XlinkX module in PD 3.1, using a non-cleavable strategy of disulfide (loss of 2.016 Da) and a validation at 1% false discovery rate [60].

### Hydrogen–deuterium exchange mass spectrometry

Hydrogen–deuterium exchange (HDX) was performed on DnaA^DI^ constructs from the following organisms: *Rh, C. bovis, S. lividans*, and *E. coli*. Additionally, HDX was performed on the *C. bovis* DnaA^DI^ (S89C) and *C. bovis* DnaA^DI^ (S89K) mutants. For each analyzed construct, 1 μl protein samples at either 20 μM (for low concentration experiments) or 600–1000 μM (for high concentration experiments) were each independently 19-fold diluted with D_2_O buffer [25 mM Tris–HCl, 150 mM NaCl, 5 mM DTT, 5% (v/v) glycerol, pD 7.5]. After each time point of the HDX the reaction was quenched at pH 2.3 by addition of 1:1 (v/v) quench buffer of 19 μl [3.5 M guanidinium hydrochloride and 0.8% (v/v) formic acid, pH 2.3] and immediately processed for MS analysis.

LC-MS with Cyclic Ion Mobility Analysis: Deuterated and control samples were analyzed as described previously [61]. Briefly, deuterated and control samples were proteolytically digested online at 15°C using an Affipro pepsin column. The cooling chamber of the HDX system was maintained at 0.0 ± 0.1°C throughout the measurements. Peptides were trapped and desalted on a VanGuard Pre-Column trap (2.1 mm × 5 mm, ACQUITY UPLC BEH C18, 1.7 μm) for 3 min at a flow rate of 100 μl/min. Elution of peptides from the trap was achieved using a 5%–35% (v/v) acetonitrile gradient with 0.1% (v/v) formic acid over 6 min at a flow rate of 100 μl/min, followed by separation on an ACQUITY UPLC HSS T3 column (1.8 μm, 1.0 mm × 50 mm). Mass spectra were acquired using a Waters SELECT SERIES Cyclic IM QTOF MS. Spectra were acquired between 50 and 2000 *m*/*z* with capillary voltage 3.0 kV, sample cone 20 V, and transfer CE ramping from 20 to 40 V. One-pass cIM was performed with a 10-ms injection, 3-ms separation, and 34-ms ejection/acquire sequence with a TW static height of 23 V and an analog-to-digital converter start delay of 13 ms and using one push per bin. The error in determining the average deuterium incorporation for each peptide was at or below ±0.5 Da, based on deuterated peptide standards.

Peptides were identified from replicate HDMS^E^ analyses of undeuterated control samples, as detailed in the text, using PLGS 3.0.3 (Waters Corporation). The peptides identified in PLGS were then filtered in DynamX 3.0 (Waters Corporation) with a minimum products per amino acid cut-off of 0.25 and at least two consecutive product ions, as outlined (see text).  Peptides with these filtering criteria were further processed by DynamX 3.0 (Waters Corporation). The software determined the relative amount of deuterium in each peptide by subtracting the centroid mass of the undeuterated peptide from that of the deuterated peptide at each time point. Deuterium levels were not corrected for back exchange and are reported as relative values [62].

### Phylogenetic analysis

Full-length sequences of DnaA were downloaded from UniProt and NCBI (Supplementary Table S4). Taxonomy follows List of Prokaryotic Names with Standing in Nomenclature (LPSN) referenced on 2 January 2026 [63]; phylogenetic relationships were based on Verma *et al*. and Val-Calvo and Vázquez-Boland [64, 65]. Taxonomic sampling was designed to be broad and representative. Within the order Mycobacteriales, we examined at least 1 and up to 5 representatives from each of the 22 genera currently recognized by LPSN where possible; every family was sampled and only 2 genera lacked accessions entirely (*Bactoderma* and *Stibiobacter*) (Supplementary Table S4). Within the class Actinomycetes, we examined at least one and up to two representatives from different genera representing each of the 54 families currently recognized by LPSN. Within the phylum Actinomycetota, we examined at least five representatives from each of the six classes currently recognized by LPSN, with as broad taxonomic diversity as possible (e.g. *Rubrobacteria* contains just one genus). In total, 159 accessions were analyzed. Full-length DnaA sequences were aligned separately at each of the three taxonomic scales using ClustalO hosted at EMBL [66, 67]. Alignments were converted and processed with Seqkit v2.11.0 and awk. For most analyses, alignments were trimmed to a majority of DnaA^DI^ spanning the seven focal residues and additional residues on either side. WebLogo v3.7.12 was used to generate sequence logos [68]. Mesquite v4.02 was used to carry out ancestral state reconstructions and to identify parallel substitutions [69].

## Results

### Structures of the *R. hoagii* DnaA^DI^ reveal a novel dimer

We initiated our structural studies on the *Mtb* DnaA^DI^. However, crystallization trials of this DnaA^DI^ failed to produce crystals. Hence, we performed homolog screening on DnaA^DI^s of related Mycobacteriales organisms and succeeded in obtaining a 1.6 Å resolution structure of the *Rh* DnaA^DI^ (the “Materials and Methods” section; and Supplementary Tables S2 and  S3). The crystallographic asymmetric unit of the structure contains two near-identical copies of the *Rh* DnaA^DI^ [root mean squared deviation (RMSD) of 0.53 Å for 70 C⍺ atoms]. In brief, the *Rh* structure consists of a single ⍺β-domain with ⍺1-⍺2-β1-β2-⍺3-β3 topology (Fig. 1B). This overall topology is similar to previously solved DnaA^DI^ structures, such as *E. coli* DnaA^DI^. In the structure, ⍺3 can be considered two helices due to a bend in its structure, but for clarity, we refer to it as a single helix. The C-terminal 20 residues could not be resolved, suggesting they are disordered and part of the flexible linker region.

Structural homology analyses using PDBeFold revealed that while the *Rh* DnaA^DI^ structure is similar overall to the four DnaA^DI^ structures solved to date, there are notable differences (Supplementary Fig. S2) [70]. The DnaA^DI^ structure with the strongest homology to the *Rh* protein is the *B. subtilis* homolog (PDB entry 4TPS); the 80 corresponding C⍺s of the two structures superimpose with an RMSD of 1.91 Å. The modestly elevated RMSD can largely be attributed to a small rotation of the *B. subtilis* DnaA^DI^ ⍺2 relative to its *Rh* counterpart. The DnaA^DI^ structures of *H. pylori, E. coli*, and *M. genitalium* exhibited RMSD values of 2.25, 2.41, and 2.92 Å for 67, 68, and 74 corresponding C⍺s, respectively. Similar to the *B. subtilis* domain, the relative orientation of ⍺2 and ⍺1 in these structures show differences relative to the *Rh* DnaA^DI^ structure. This may be explained, in part, by the *Rh* DnaA^DI^ ⍺1–⍺2 loop, which is longer than its analog in the other four structures. Other differences include the presence of a loop instead of β1 in the *H. pylori* DnaA^DI^ and the addition of a helix following β3 in the *M. genitalium* DnaA^DI^.

The most salient and unexpected feature of the *Rh* DnaA^DI^ structure is the presence of a head-to-head dimer in the crystal (Fig. 1C and D). The interface of this dimer is supported by three elements; an ⍺3–⍺3′ (where ' indicates other subunit of the dimer; E64, R68, and their protomeric counterparts) salt bridge network (herein called the “polar zipper”), a β3–β3′ water-mediated IMS, and a cluster of six hydrophobic side chains, including F49 and F49′, contributed by residues from both subunits (the “hydrophobic anchor”) (Fig. 1E). This dimer is notably distinct from that proposed for the *E. coli* DnaA^DI^, which was suggested to be formed via contacts from W6 (located on the ⍺1 helix), β1, and possible contacts from β2 and the N-terminus of β3 [29, 71]. It was possible that the dimer observed in our structure is a crystallographic artifact. Hence, we obtained a second *Rh* DnaA^DI^ crystal form, with unique crystallographic packing, using a different crystallization condition (Fig. 1D and Supplementary Table S2). This structure was solved to 3.0 Å resolution and revealed a dimer identical to that observed in our other structure. Indeed, the dimeric structures can be superimposed with an RMSD of 0.522 Å for 150 aligned C⍺ atoms.

### Biochemical tests of the DnaA^DI^ dimer

The conservation of the dimer between the two *Rh* DnaA^DI^ crystal forms suggests its possible biological relevance. To test DnaA^DI^ dimer formation further, we designed a collection of orthogonal biochemical assays. To this end, we analyzed our crystal structures to identify amino acid substitutions that would disrupt the putative dimer. The tight packing of the intermolecular β3–β3′ sheet places steric limitations on the side chain inhabiting the central position of the strands. Thus, we called this residue position, within the IMS, the “steric selection site.” In the WT *Rh* DnaA protein, this position is occupied by glycine. We reasoned that mutation of this site to a residue with a large, polar side chain would disrupt dimer formation. Thus, we generated, expressed, and purified the *Rh* DnaA^DI^ (G87K) protein.

**Figure 2.**
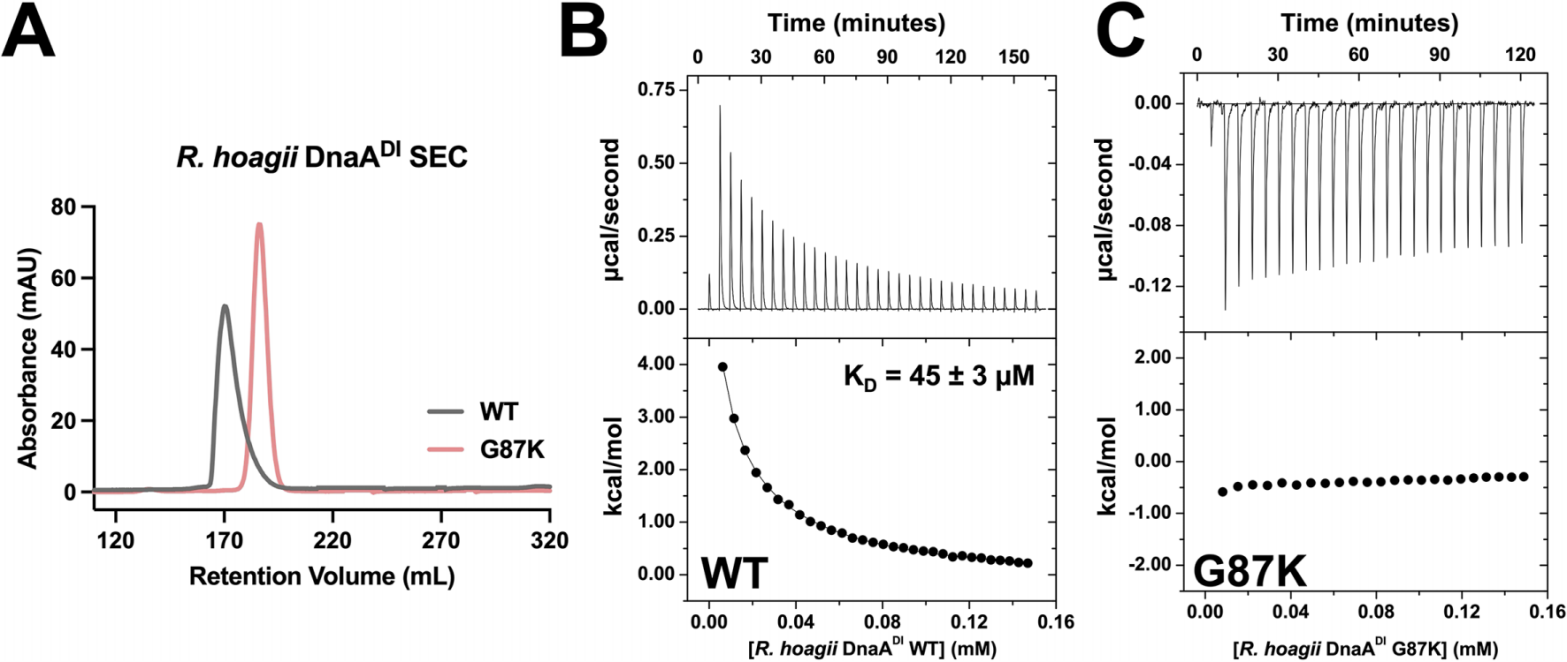
The *Rh* DnaA^DI^ forms a dimer in solution. (**A**) SEC analyses of WT and G87K *Rh* DnaA^DI^ proteins. Consistent with the structural dimer, the WT species exhibits a decreased retention time on the SEC column relative to its G87K counterpart. Proteins were eluted at 1 ml/min. (**B**) hdITC thermogram of the *Rh* DnaA^DI^ WT. (**C**) hdITC thermogram of the *Rh* DnaA^DI^ G87K mutant. For comparison, the integrated heat trace is presented on the same absolute scale as its WT counterpart, shifted by ∼2 kcal/mol.

To assess for the formation of oligomers in solution, SEC experiments were performed on the WT *Rh* DnaA^DI^ and DnaA^DI^ (G87K) proteins at 0.75 mg/ml. Consistent with the formation of a higher order species, the WT DnaA^DI^ protein exhibited decreased retention time on the column relative to DnaA^DI^ (G87K) (Fig. 2A). In addition, while the DnaA^DI^ (G87K) mutant protein elution profile is symmetric, consistent with the migration of a uniform protein population, the WT DnaA^DI^ elution profile exhibits a tail, suggesting an equilibrium population of monomer and dimer states. We noted that the *Mtb* DnaA^DI^ also contains a glycine at the steric selection site and indeed similar SEC profiles were obtained for the WT *Mtb* DnaA^DI^ and steric selection mutant, *Mtb* DnaA^DI^ (G97K), suggesting that the *Mtb* DnaA^DI^ forms similar oligomers as the *Rh* protein (Supplementary Fig. S3).

As an additional test of the solution oligomeric state of the *Rh* DnaA^DI^, we developed a homodimer-dissociation isothermal titration calorimetry (hdITC) assay. In hdITC, a concentrated protein solution is injected into buffer and the resultant heat exchange is monitored. The protein, which forms homodimers in the concentrated syringe solution, will dissociate upon dilution in the cell, releasing or absorbing heat in the process. Each stepwise injection is expected to generate a smaller heat signal than the previous as a consequence of increasing protein concentration in the cell. The resultant thermogram can be fit to a decay function (see the “Dissociation” function as implemented in Origin 7.0) to extract thermodynamic information. This method has been successfully employed to quantify the homo-dissociation of several macromolecules, including CK2β, which was analyzed in the presence of thermally destabilizing fragments [72–76]. hdITC was performed on both WT *Rh* DnaA^DI^ and DnaA^DI^ (G87K). The resultant hdITC thermogram for the WT DnaA^DI^ protein exhibited decay-behavior characteristic of homooligomer-dissociation. Non-linear regression fitting of the data to the Origin dissociation function yields a K_D_ of ∼45 µM (Fig. 2B). In contrast, the thermogram for the DnaA^DI^ (G87K) protein exhibited no decay behavior (Fig. 2C).

To test the relevance of the structurally observed hydrophobic anchor and polar zipper dimerization elements, we expressed and purified two additional *Rh* DnaA^DI^ mutant proteins: *Rh* DnaA^DI^ (F49T), to disrupt the hydrophobic anchor; and *Rh* DnaA^DI^ (E64A), to abolish the ⍺3–⍺3′ polar zipper. Consistent with our structural model, both F49T and E64A *Rh* mutant constructs demonstrated increased retention time on an SEC column relative to their WT counterpart (Supplementary Fig. S4A). The F49T construct elution profile was indistinguishable from that of the G87K mutant, suggesting the F49T mutation completely abolishes dimerization. Like the F49T and G87K constructs, the elution profile of the E64A mutant protein was consistent with disruption of the solution-state dimer interaction. However, unlike the F49T and G87K mutants, the E64A SEC profile was not symmetric and demonstrated a clear tail. These observations suggest that the E64A mutation disrupts, but does not completely abrogate, *Rh* DnaA^DI^ dimerization. Consistent with this, hdITC experiments revealed that the E64A protein exhibited an affinity of dimerization ∼10× weaker than that of the WT protein (Supplementary Fig. S4B). Notably, CD spectra collected for WT and the three dimer-disruption mutants overlapped. Therefore, differences in SEC and hdITC results observed between the WT and mutant proteins are not attributable to mutation-induced conformational changes or misfolding (Supplementary Fig. S4C).

### Structures and biochemical analyses of DnaA^DI^s from select Actinomycetes

**Figure 3.**
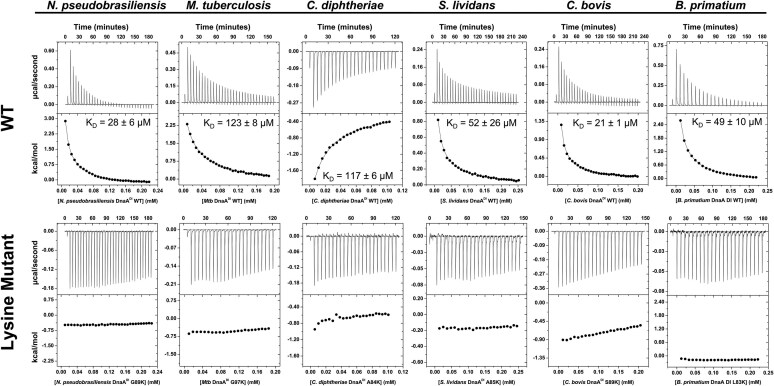
The *Rh* DnaA^DI^ dimer is conserved across members of the Actinomycetes class and can be supported by non-glycyl residues at the steric selection position. In the upper row, representative hdITC thermograms for select Actinomycetes DnaA^DI^ proteins are presented. Reported K_D_ are the average of two or three experimental replicates with error given by the standard deviation of the measurements. In the lower panel, the hdITC thermograms are presented for corresponding steric selection site lysine mutants. For comparison, corresponding WT and lysine-mutant integrated heat traces are presented on the same absolute scale with appropriate vertical shifts.

Having provided evidence of the solution state relevance of the *Rh* DnaA^DI^ dimer, we next considered the broader taxonomic significance of the interaction. A cursory analysis of DnaA^DI^ sequences across taxa hinted at conservation of the dimer in the class Actinomycetes. Specifically, amino acids with small side chains (i.e. glycine, alanine, and serine) appeared ubiquitously at the steric selection site, a necessary condition for dimerization. Less commonly observed were amino acids with slightly larger side chains at the steric selection site, including valine, leucine, and isoleucine. To test whether the DnaA^DI^ of other Actinomycetes organisms formed dimers and to determine which amino acid residues could be accommodated at the steric selection site, we performed hdITC experiments on DnaA^DI^ proteins from select members of the class. These include the DnaA^DI^ proteins, with steric selection site identity noted, from the following organisms: *Mtb* and *N. pseudobrasiliensis*, encoding glycine; *C. diphtheriae* and *S. lividans*, encoding alanine; *C. bovis*, encoding serine; and *B. bifidum* encoding leucine. For each construct, we generated a dimerization-disruption mutant in which the steric selection position was mutated to lysine. The hdITC thermograms for each WT DnaA^DI^ protein exhibited decay-behavior characteristic of homooligomer-dissociation, suggesting that these DnaA^DI^s all form multimers. In contrast, proteins in which the steric selection site was mutated to lysine demonstrated no such decay behavior, suggesting that the dimers formed by these proteins may exhibit similar interfaces to that of the *Rh* DnaA^DI^ dimer (Fig. 3). Notes: (i) Experiments were also performed on the *B. bifidum* DnaA^DI^. However, due to experimental complications, these data are considered separately (Supplementary Fig. S5) [77–79]; (ii) In contrast to all other WT DnaA^DI^ constructs, which exhibited endothermic heats of dilution, the WT *C. diphtheriae* construct reproducibly exhibited exothermic heats of dilution. These data suggest that the thermodynamics of *C. diphtheriae* DnaA^DI^ dimerization differ from its counterparts, though we have no current explanation for this observation.

**Figure 4.**
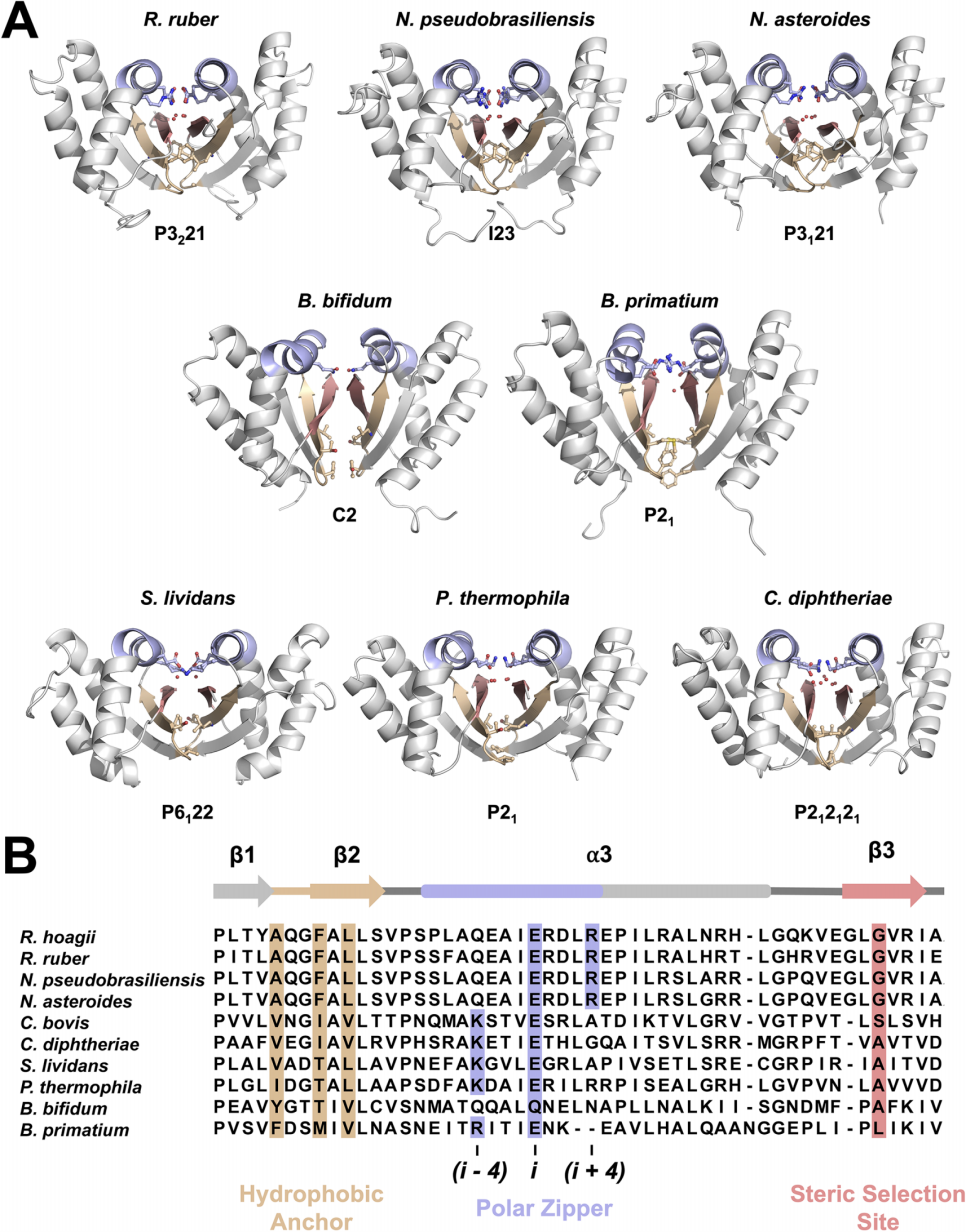
Structures of the conserved DnaA^DI^ dimer from select Actinomycetes organisms. (**A**) The crystal structures of additional Actinomycetes DnaA^DI^s were solved revealing a conserved dimerization interface. Conserved dimerization elements, including the polar zipper, intermolecular β-sheet, and hydrophobic anchor, are presented in light blue, salmon, and wheat, respectively. Residues and atoms involved in critical interfacial interactions are shown in ball-and-stick representation. The crystallographic space groups for each structure are included under the structure. (**B**) Alignment of DnaA^DI^ proteins examined in this study with corresponding secondary structure diagram. Alignment generated with ClustalOmega. Dimerization elements are indicated by color as in the 3D-models.

While our hdITC data suggest that DnaA^DI^ dimerization may be conserved across the Actinomycetes, the *Rh* DnaA^DI^ structure does not provide an explanation for how serine, leucine, and other non-glycyl residues can be accommodated at the steric selection site of these dimers. Indeed, molecular modeling suggests that even alanine could not be accommodated at the steric selection site in the *Rh* DnaA^DI^ dimer context. Thus, to understand how differences in the amino acid composition of dimerization elements may impact the specific molecular architecture of the DnaA^DI^ dimer interface, we solved DnaA^DI^ crystal structures of nine additional organisms, representing four biological orders (Fig. 4A and Supplementary Fig. S6A and B). Consistent with our biochemical data, all these structures exhibited the same general dimer, conserving the three dimerization elements observed in the *Rh* DnaA^DI^ structures (Fig. 4B). The first element is the hydrophobic anchor, a collection of hydrophobic side chains contributed by residues in β2 and the β1 – β2 loop. These residues form a densely packed, water-occluding core at the bottom of the dimerization interface. We note that, despite its characterization as a polar amino acid, threonine functions as a hydrophobic anchor residue in some DnaA^DI^s; in these dimers, the threonine hydrophobic methyl group points inward, toward the hydrophobic anchor, while its hydroxyl group is directed outward toward the solvent (Supplementary Fig. S7A). The second element is the polar zipper, which is comprised of one or two polar residues on ⍺3 that make side chain contacts with their subunit partner(s). Most commonly, an acidic residue, generally glutamate, occupies a central position, ***i***, that forms a salt bridge with a basic residue (lysine or arginine) located at position (***i + 4***) or (***i − 4***). Thus, three positions in DnaA^DI^ can be utilized in the polar zipper interaction (Supplementary Fig. S7B). In some *Bifidobacteriales* organisms, such as *B. bifidum*, a distinct mechanism of polar zipper formation is observed whereby a glutamine or asparagine residue forms a bidentate hydrogen bond with its protomeric counterpart (Supplementary Fig. S7A). The third dimerization element is the β3–β3′ IMS, which harbors the steric selection site. In most structures, the IMS is a solvent-mediated interaction. Specifically, water molecules line the internal surface of the dimerization interface, acting as simultaneous hydrogen-bond acceptors and donors for opposing β3-backbone amides and carbonyls, respectively. However, the β3–β3′ interaction is mediated by direct backbone contacts in some dimers, such as the *B. bifidum* DnaA^DI^, suggesting that multiple chemical mechanisms are consistent with IMS formation.

While all DnaA^DI^ dimers reported here encode the same dimerization elements and share similar interface surface areas and affinities of dimerization (Supplementary Table S5), comparisons of the structures reveal variations in the quaternary organization of their constituent subunits. In particular, notable differences (offsets) in the angular and translational arrangement of subunits are observed across the DnaA^DI^ dimers. These offsets in the interaction register and angle appear to provide the primary basis for molecular accommodation of larger, non-glycyl residues at the steric selection position (Supplementary Fig. S8). Specifically, subunit offsets expand the selection site “steric envelope,” rendering larger amino acids permissible at the interface. Changes in interaction register are readily apparent upon analysis of the β3–β3′ IMS (Supplementary Fig. S8). For dimers encoding glycine at the steric selection site, a face-to-face configuration of steric selection residues is adopted. In contrast, for dimers encoding non-glycyl residues at the selection site, steric selection site residues are displaced laterally, adopting a staggered conformation.

Changes in the interaction angle of the dimer protomers are also evident and can be quantified by considering the β3–β3′ intersection angle (Supplementary Fig. S8, and the “Materials and Methods”  section). Of particular note is the uniquely large β3–β3′ interaction angle demonstrated in our *Bifidobacteriales* structures. In these dimers, the IMS interaction angle is ∼45°–55° from horizontal. In contrast, the largest such angle in a non-*Bifidobacteriales* dimer is ∼35°, as seen in the *C. diphtheriae* assembly; in the *N. asteroides* dimer, this angle is only ∼10° and is similarly small in other glycine-encoding dimers. The large IMS interaction angle exhibited by the *Bifidobacteriales* dimers imposes increased lateral separation between the selection site side chain and the opposing β3-strand. The increased separation afforded by this interaction appears to explain the otherwise rare presence of valines, isoleucines, and leucines at the steric selection site of *Bifidobacteriales* DnaA^DI^ proteins.

### Crosslinked *C. bovis* DnaA^DI^ data support dimerization model

**Figure 5.**
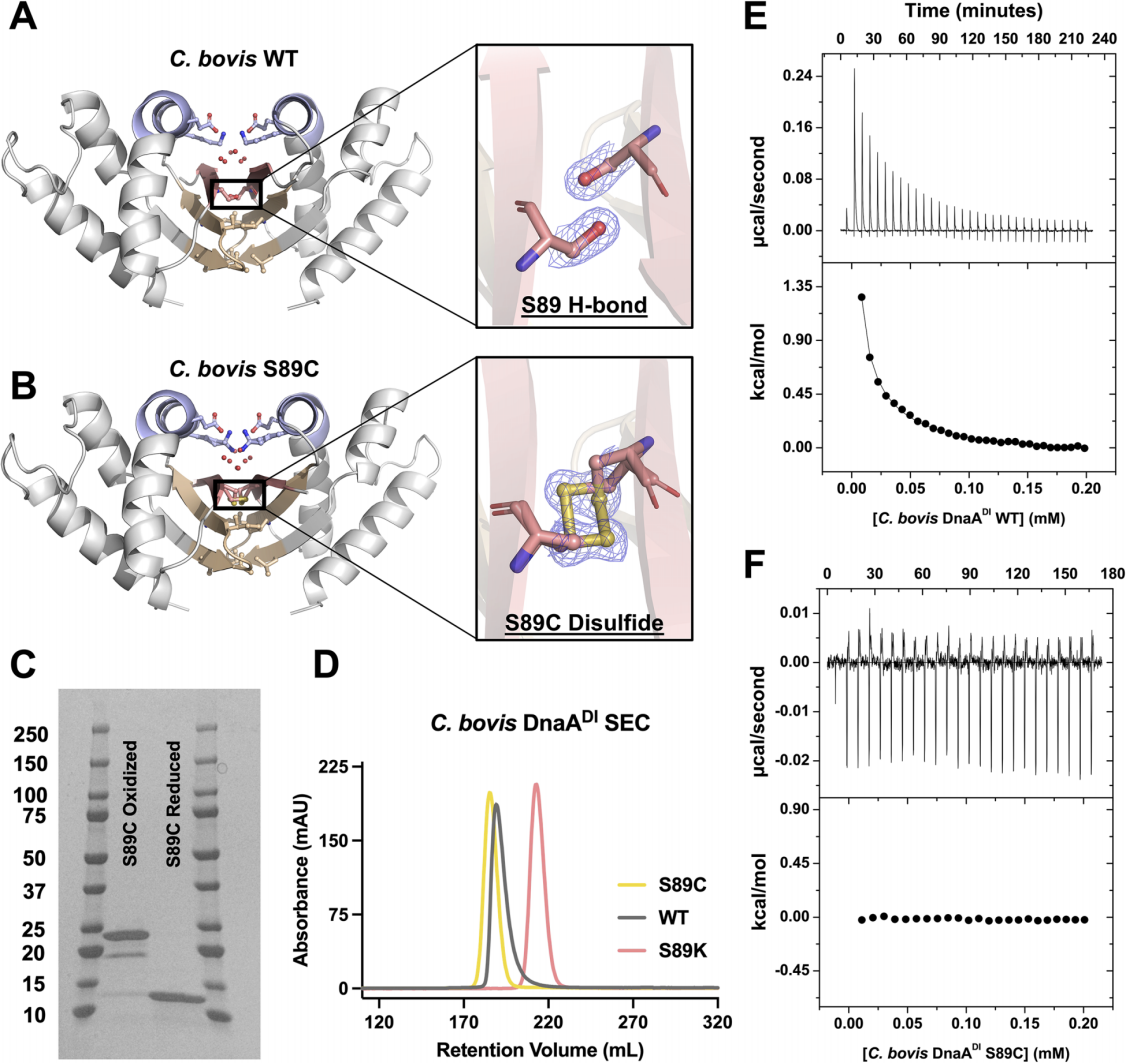
The *C. bovis* disulfide-linked DnaA^DI^ dimer supports the structural dimer model. (**A**) Crystal structure of the *C. bovis* DnaA^DI^ dimer. Dimerization elements are presented as described above. In zoom, the steric selection site S89 residue is shown making a strong hydrogen bond to its S89′ protomeric counterpart. 2*m*F_O_ – DF_C_ mesh density (contoured at 2.0 σ with a 1.6 Å carve radius) is included around the side chain. (**B**) Crystal structure of the disulfide-linked *C. bovis* DnaA^DI^ (S89C) dimer. The disulfide link, captured in two distinct conformations, is presented in zoom with 2*m*F_O_ – DF_C_ mesh density (contoured at 2.0 σ with a 1.6 Å carve radius) shown around the cystine linkage. (**C**) SDS–PAGE gel of the *C. bovis* DnaA^DI^ (S89C) mutant run under oxidizing or reducing conditions. The oxidized species migrates with an apparent molecular weight of ∼25 kDa, consistent with a dimer; the reduced species runs with an apparent molecular weight of ∼12 kDa, consistent with a monomer. (**D**) SEC analyses of the *C. bovis* WT, S89C, and S89K DnaA^DI^ proteins. Gel phase distribution coefficient analysis revealed the S89K and S89C species migrate with molecular weights of 16 and 31 kDa, respectively, consistent with a monomer–dimer relationship. The WT species has a similar retention volume as the S89C species and migrates with an apparent molecular weight of 28 kDa, consistent with a dimer. (**E**) hdITC analysis of the WT *C. bovis* DnaA^DI^. (**F**) hdITC analysis of the *C. bovis* DnaA^DI^ (S89C) species. Unlike the WT species, the S89C construct demonstrates no apparent heat of dissociation. These data suggest the heat liberated in the WT assays can be attributed to dissociation of a multimeric (presumably dimeric) species. The WT hdITC trace is reproduced from Fig. 3.

Interestingly, the *C. bovis* DnaA^DI^ contains a serine, S89, at the steric selection position. Our structure revealed that its accommodation at the dimerization interface is achieved not only by offsets in the β3–β3′ interaction angle and register, but also by the formation of a hydrogen bond between S89 and its protomeric counterpart, S89’ (Fig. 5A). We noted that the interaction geometry of the S89 and S89’ residues would appear to accommodate a disulfide bond. Thus, to test whether a *C. bovis* DnaA^DI^ (S89C) mutant might form disulfide crosslinks, we expressed and purified the protein under non-reducing conditions. The protein readily formed disulfide-linked dimers in the presence of a Cu^2+^ catalyst, as confirmed by a crystal structure, SDS–PAGE, and MS (Fig. 5B and C; and Supplementary Fig. S9). In the *C. bovis* DnaA^DI^ (S89C) structure, clear density for the disulfide, which adopts two distinct conformations, was observed (Fig. 5B). The crosslink was minimally disruptive to the local and global dimer structure, evidenced by an RMSD of 0.35 for all C⍺ atoms of the WT and S89C dimers. The formation of the crosslinked species provides additional support for the solution-state relevance of the dimer and may serve as a new tool for studying Actinomycetes DnaA^DI^ dimers.

We next analyzed the crosslinked *C. bovis* DnaA^DI^ (S89C) protein by SEC and hdITC (Fig. 5D–F). Our hdITC data showed that, in sharp contrast to the WT species, but like the S89K species, the crosslinked DnaA^DI^ (S89C) did not demonstrate a homodissociation decay isotherm, consistent with the covalent nature of the DnaA^DI^ (S89C) dimer, which cannot dissociate (Fig. 5E and F). SEC was performed on the WT, crosslinked S89C, and S89K *C. bovis* DnaA^DI^ species at 2 mg/ml. Gel phase distribution coefficient analysis of DnaA^DI^ (S89K) and crosslinked DnaA^DI^ (S89C) species (which both exhibited symmetric elution profiles) revealed a monomer and dimer (16 and 31 kDa, respectively), while the WT construct migrated with an apparent mass of 28 kDa, consistent with the migration of an equilibrium dimer (Fig. 5D; and Supplementary Fig. S10). The small decrease in the apparent molecular weight of WT relative to S89C protein is most likely due to a minor WT monomer population present at equilibrium.

As hdITC and SEC proved to be powerful tools for analyzing the dimerization of Actinomycetes DnaA^DI^ proteins, we next used these techniques to study the purported *E. coli* and *H. pylori* DnaA^DI^ dimers [29, 45]. We note that the dimerization of the *H. pylori* DnaA^DI^ is disputed, with different studies providing opposing conclusions [35, 45]. We were unable to detect dimerization for either the *E. coli* or *H. pylori* DnaA^DI^ under our experimental conditions (Supplementary Fig. S11A–C). Hence, these DnaA^DI^s do not appear to form similar dimers to those formed by their Actinomycetes counterparts. Indeed, our analyses show that all four DnaA^DI^ proteins for which structural information is available appear to encode elements incompatible with the steric requirements of the Actinomycetes dimerization-mode (Supplementary Fig. S2). Specifically, the DnaA^DI^ proteins from *E. coli, B. subtilis*, and *H. pylori* encode arginine or lysine at the steric selection position, and the *M. genitalium* DnaA^DI^ encodes an additional helix that occludes the relevant interaction surface. Thus, further studies will be needed to clarify the molecular basis for any multimeric interactions of the DnaA^DI^s of distinct bacterial phyla.

### Phylogenetic and evolutionary analyses of DnaA^DI^s

**Figure 6.**
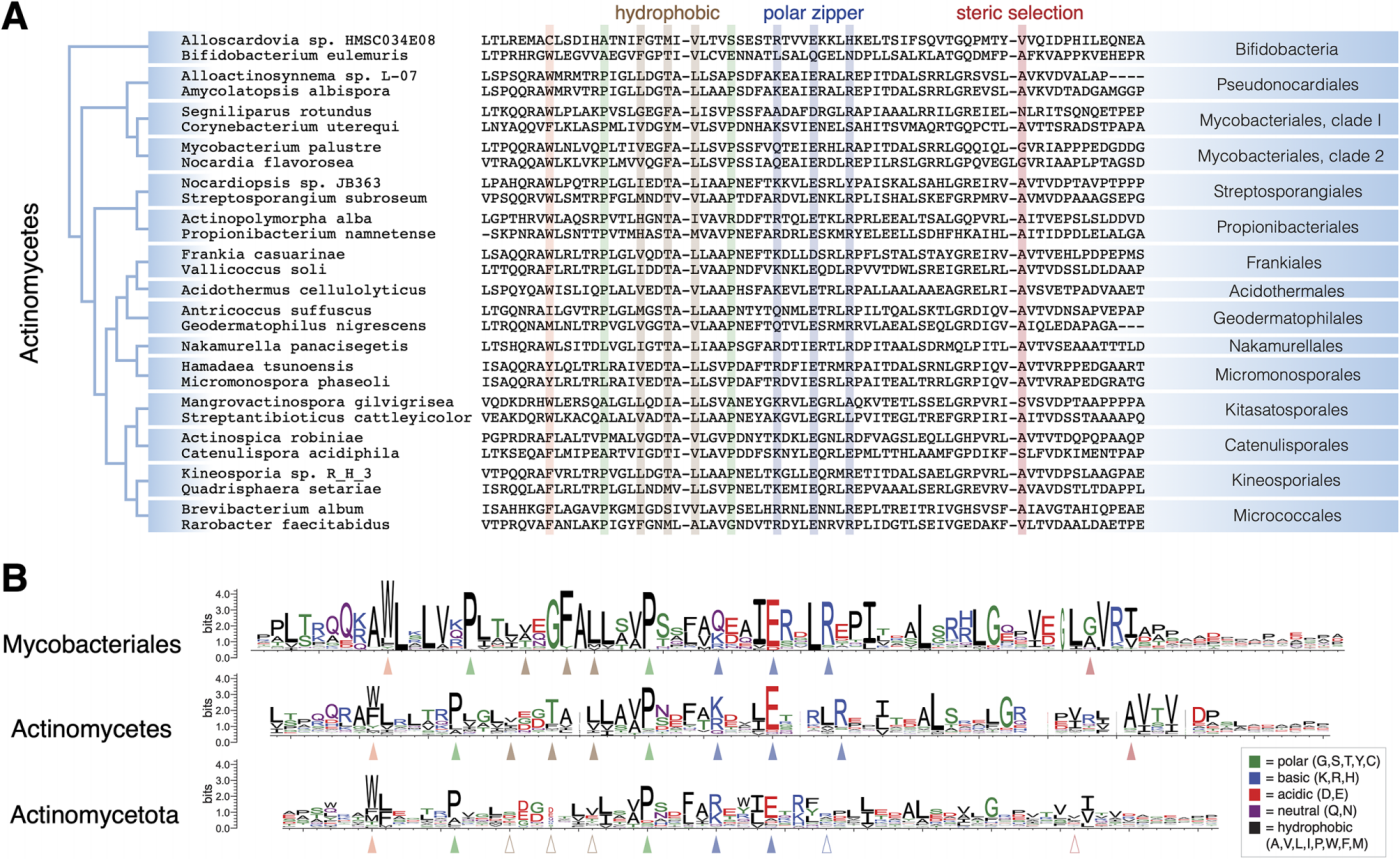
Conservation of the DnaA^DI^ dimer. (**A**) Aligning sequences representing taxonomic diversity reveals broad conservation of key residues for dimerization and tertiary structure. Alignment representing every order within the class Actinomycetes. See Supplementary Figs S12–S14 for deeper taxonomic sampling representing every family. The seven focal residues critical for dimerization are highlighted, along with three residues that may contribute to the tertiary structure of domain I. Independent alignments from additional species at closer and more distant scales of divergence are shown in Supplementary Figs S12–S14. (**B**) Sequence logos at three scales of divergence. Colors reflect side chain chemistry (see inset); lengths differ due to insertions and deletions present in independent alignments. In order to reduce ascertainment bias, the logo for the phylum Actinomycetota does not include sequences from Actinomycetes. The seven focal residues critical for dimerization and three residues relevant for tertiary structure in Mycobacteriales and Actinomycetes are indicated with arrowheads colored to match the highlighting in panel (A). Possible functional equivalents in other classes of the phylum Actinomycetota are not experimentally supported but are based on a combination of alignment and side chain chemistry; open arrowheads indicate orthologous positions where side chain chemistry is not well conserved.

Our combined structural and biochemical data revealed that all examined Actinomycetes DnaA^DI^ proteins form the same novel and previously unknown dimer. To determine the taxonomic breadth and evolution of this oligomer, we performed a comprehensive phylogenetic study to delineate the extent to which the key dimerization elements are conserved among taxa. Informed by our structural data, we selected seven residues as the foci of our analysis: the IMS steric selection site; three residues of the hydrophobic anchor; and the three residues that contribute to the polar zipper [the residues of ⍺3 appropriately positioned for interfacial contact, residues ***i*** and (***i* ± 4**)]. We examined phylogeny-informed alignments of the DnaA^DI^ from 159 species across three scales of evolutionary divergence: within the order Mycobacteriales, the class Actinomycetes, and the phylum Actinomycetota. Figure 6A shows a summary alignment of DnaA^DI^ across the class Actinomycetes. The full alignment and alignments at other scales are shown in Supplementary Figs S12–S14; taxa, sources, and accessions are listed in Supplementary Table S4.

Our analyses show that the seven focal residues are well conserved throughout the class Actinomycetes, although none perfectly. Most substitutions involve functionally similar side chains, with BLOSUM scores overwhelmingly 0 or positive. In addition, placing substitutions into a phylogenetic context indicates that several specific substitutions evolved multiple times, while other substitutions involving side chains of similar size and charge are not observed among the taxa sampled. Conservation within the DnaA^DI^ is not limited to the seven residues. Additionally conserved sites include two nearby prolines, a tryptophan, and a leucine (Fig. 6A). Most of these additional conserved sites likely support domain tertiary structure. These observations suggest that negative selection acts to maintain residues critical for dimerization and the tertiary structure of the DnaA^DI^ throughout the radiation of the class Actinomycetes. In contrast to the class Actinomycetes, five of the seven focal residues are not well conserved across other classes of the phylum Actinomycetota (Fig. 6B and Supplementary Figs S13 and S14). Only the first and second residues of the salt bridge are well conserved. While a detailed phylogenetic analysis of differences among Actinomycetota classes was not attempted, it is clear that only a minority of the focal residues are broadly conserved throughout the phylum (Supplementary Data). Thus, these analyses show that the DnaA^DI^ dimer elements are broadly conserved across the Actinomycetes but do not extend to the entire Actinomycetota phylum.

Considering each of the seven focal residues individually reveals distinct degrees of conservation throughout the class Actinomycetes (Fig. 6B). Four are overwhelmingly represented by a single side chain throughout the class: threonine in the center of the hydrophobic core, glutamic acid and arginine at the ***i*** and (***i + 4***) positions of the salt bridge, and alanine at the steric selection site. Within the order Mycobacteriales, threonine in the hydrophobic core is widely replaced by phenylalanine and alanine in the steric selection site by glycine, but this is not the case in other orders. Our experimental data showed that the steric selection site can tolerate glycine, alanine, serine, and leucine. Likely, valine and isoleucine can also be accommodated due to their steric similarity to leucine. One of these six permissible residues is present at the steric selection site in 96.6% (28/29) of accessions examined in the order Mycobacteriales and in 95.9% (93/97) of accessions in the class Actinomycetes.

Two other focal residues are somewhat less conserved within the *Actinomycetes* but limited to a narrow range of possibilities in nearly all sampled accessions: the third position in the hydrophobic core is dominated by leucine and valine, while the (***i - 4***) position in the salt bridge is largely limited to lysine, arginine, and glutamine. The first position in the hydrophobic core is the most variable: valine, leucine, methionine, isoleucine, histidine, and alanine are all common, and transitions among residues are frequent in comparison with the other six focal positions. Notably, these residues are all compatible with existing within the hydrophobic core. Supplementary Table S6 enumerates the range of amino acids at each position among the sampled taxa. Ancestral state reconstruction indicates the likely identity of six of the seven focal residues in the ancestor of the Actinomycetes; only the first position in the hydrophobic core is ambiguous. The other six are reconstructed as Thr, Leu, Lys, Glu, Arg, and Ala.

### Hydrogen–deuterium exchange mass spectrometry of DnaA^DI^

As our biochemical and bioinformatic studies showed a conserved mechanism of DnaA^DI^ dimerization across the Actinomycetes, we next sought to probe the consequences of dimerization on DnaA^DI^ structural dynamics using HDX-MS [80, 81]. Guided by biochemical and structural analyses of DnaA^DI^ constructs differing at the β3–β3′ interface, including, in the case of the *E. coli* DnaA^DI^, substitutions that alter steric compatibility, we performed hydrogen–deuterium exchange mass spectrometry (HDX-MS) on the *Rh, C. bovis, S. lividans*, and *E. coli* DnaA^DI^ constructs at two protein concentrations (low, 1 µM and high, 30–50 µM) to mimic the monomer and dimer states. We first compared baseline HDX profiles of all DnaA^DI^ constructs at high concentration through a qualitative analysis of their relative percent deuterium uptake and found broadly similar dynamic behavior across the four proteins (Supplementary Fig. S15). Importantly, peptides covering α3, β3, and β1–β2, the regions identified in the crystal structures as forming the core dimer interface, were observed to have low HDX across all constructs, consistent with these being intrinsically stable elements of the dimer core of DnaA^DI^.

**Figure 7.**
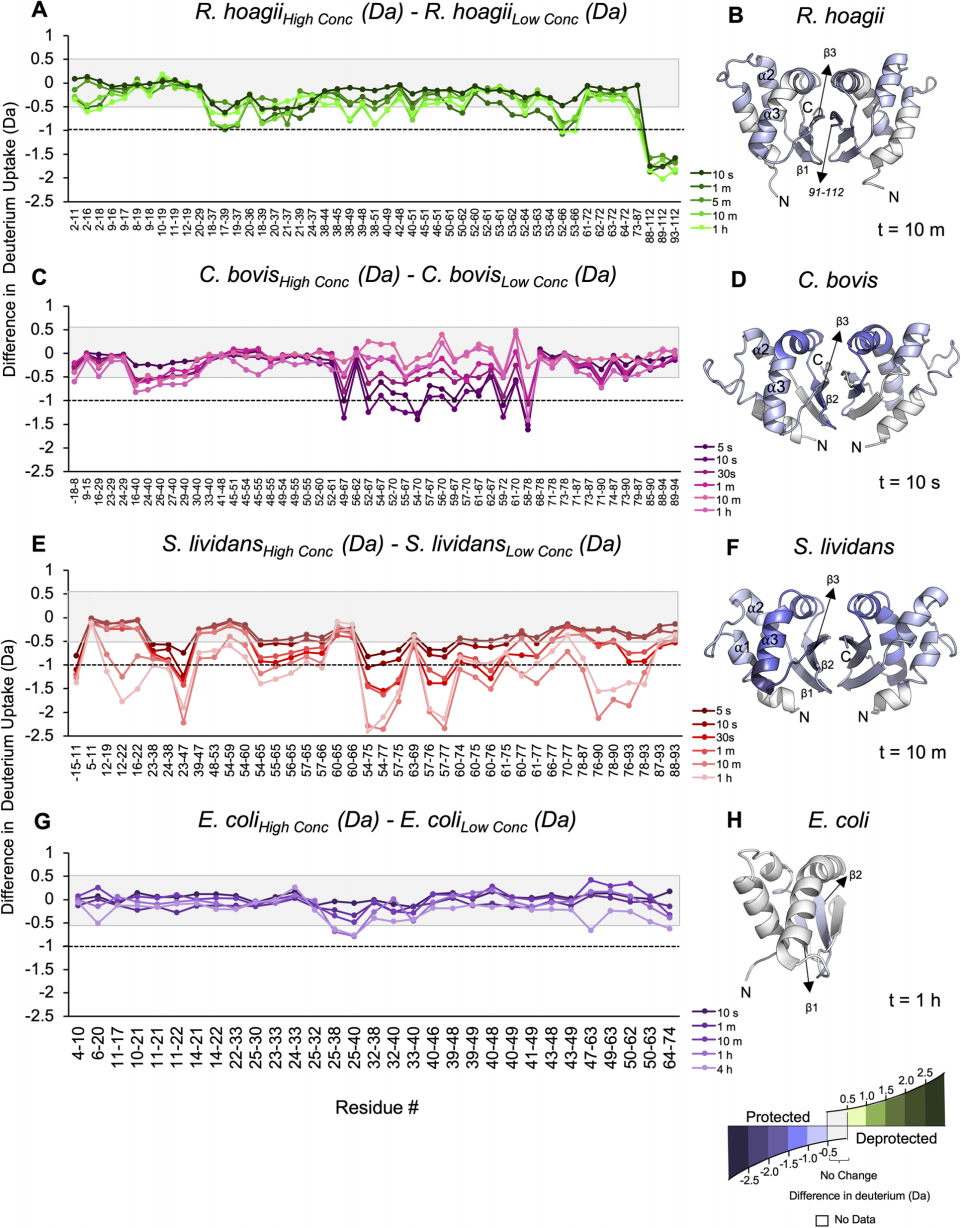
HDX-MS reveals concentration-dependent DnaA^DI^ dimer interface in Actinomycetes DnaA^DI^s. Deuterium difference plots showing the relative deuterium incorporation of DnaA^DI^ at higher concentration for (**A**) *Rh*, 1 mM; (**C**) *C. bovis*, 830 µM; (**E**) *S. lividans*, 684 µM; and (**G**) *E. coli*, 925 µM minus the relative deuterium incorporation of its low concentration counterpart (20 µM). Regions of deprotection (shades of green) and protection (shades of purple) above the 0.5 Da significant threshold at respective labeling time points are mapped onto the structure of (**B**) *Rh, t* = 10 min; (**D**) *C. bovis, t* = 10 s; (**F**) *S. lividans, t* = 10 min; and (**H**) *E. coli, t* = 1 h (PDB entry 2E0G). Black dotted line is drawn in each panel to show the demarcation of 1 Da difference. HDX-MS experiments were performed at least twice (Supplementary Table S7). The HDX-MS data used to create this figure can be found in Supplementary Table S7.

Next, we examined the comparative HDX of the induced monomer and dimer states of various DnaA^DI^ constructs (Fig. 7A–H). Overall, magnitudes of the differences in deuterium incorporation between the low and high protein concentrations varied among the four constructs. The strongest concentration-dependent protection was observed for *S. lividans* DnaA^DI^, whereas *C. bovis* and *Rh* DnaA^DI^ exhibited comparable differences in HDX. In contrast, we observed mild decreases in deuterium incorporation at the higher protein concentration for *E. coli* DnaA^DI^ residues corresponding to α2 helix, β1, and β2. This protection is consistent with previous NMR analyses that supported an *E. coli* DnaA^DI^ dimer involving β1 (and possibly β2). However, our data showed no significant protection for α1, which was noted as the main dimerization region for the *E. coli* DnaA^DI^ dimer (Fig. 7G and H) [29]. Notably, the W6 proposed to support *E. coli* DnaA^DI^ dimerization is only partially solvent exposed, with significant surface area of the side chain buried, possibly explaining the weak nature of the dimer. It is also possible that W6 may be important to stabilization of the *E. coli* DnaA^DI^ tertiary structure in addition to its quaternary structural roles. Nonetheless, these collective data indicate that any putative *E. coli* DnaA^DI^ dimer does not resemble those of its Actinomycetes counterparts.

HDX-MS analysis for the *Rh* DnaA^DI^ showed decreased deuterium uptake for the sample labeled at high concentration across multiple regions, including the C-terminal region of the α1 helix, the α2 and α3 helices, and the β1, β2, and β3 strands (Fig. 7A and B). The C-terminal region (residues 93–112) of the *Rh* DnaA^DI^ was not resolved in our experimental electron density map. At low protein concentration, residues 88–112, which encompass the flexible C-terminal region (residues 93–112) and a small segment (residues 88–90) of β3, exhibited high relative deuterium incorporation (∼50%–60%) from the earliest labeling time points, consistent with a weakly H-bonded structure and highly dynamic state (Supplementary data and Supplementary Table S7). At high protein concentrations, the same region displayed reduced deuterium incorporation (∼40%–50%), suggesting this region (or part of it, such as the β3 segment) becomes ordered and stabilized upon dimerization.

Next, as observed for the *Rh* DnaA^DI^, HDX-MS analysis of *C. bovis* DnaA^DI^ (Fig. 7B and C) revealed decreased deuterium uptake at high protein concentration across multiple regions (though, in contrast, excluding β1). We then compared the deuterium uptake of the WT *C. bovis* DnaA^DI^ to that of the S89K and S89C constructs. The covalently modified S89C variant at both low and high concentrations displayed a greater decrease in HDX across regions α1, α2, β2, α3, and β3 when compared to the WT *C. bovis* DnaA^DI^ at a high concentration (Supplementary Fig. S16A and B), consistent with a stabilized dimeric state that is independent of protein concentration. The S89K variant showed no concentration-dependent differences in deuterium uptake (Supplementary Fig. S16C and D). In contrast to the S89C construct, the S89K variant exhibited substantially higher deuterium incorporation, consistent with a more dynamic, monomeric conformation, comparable to that of the wild-type DnaA^DI^ at low concentration.

Consistent with the HDX profiles observed for the above DnaA^DI^ constructs, HDX-MS analysis of *S. lividans* DnaA^DI^ (Fig. 7E and F) showed decreased deuterium incorporation for the high protein concentration across similar regions as *Rh* and *C. bovis* but with a greater magnitude of protection from HDX. These regions included the α1–α3 helices and the β1–β3 strands.

Taken together, these HDX-MS data show three main regions that undergo concentration-dependent stabilization in the DnaA^DI^ of proteins from *Rh, C. bovis*, and *S. lividans*: the α3 helix, the β3 region, and the β1–β2 region. These regions correspond, respectively, to the polar zipper, the IMS, and the hydrophobic anchor defined by our structural analysis. These data illustrate the concordance between the solution-state HDX-MS data and the conserved dimerization architecture.

## Discussion

The initiator, now called DnaA, was anticipated over 60 years ago in the seminal work of Jacob *et al*. [6]. As the gatekeeper of DNA replication in bacteria, DnaA has been a central object of biochemical research since its initial purification in 1982 [82]. During this time, numerous studies have demonstrated the indispensable role of the DnaA^DI^ in orisome assembly and its regulation. In particular, a wealth of genetic and biochemical data suggest that the DnaA^DI^ is important in the stepwise and cooperative assembly of the orisome complex. Despite this, a stunning paucity of structural information exists to clarify the molecular nature of the mechanisms involved. To characterize this essential domain, we performed a battery of structural, biochemical, and phylogenetic studies. These investigations revealed a conserved mechanism of DnaA^DI^ dimerization across the Actinomycetes and the molecular details behind this interaction.

In particular, the DnaA^DI^ structures presented here exhibit a unified dimerization mode, supported by three multimerization elements that we termed the polar zipper, IMS, and hydrophobic anchor. To reconstruct how widespread this mode of DnaA^DI^ dimerization is likely to extend, we examined evolutionary conservation and change of the DnaA^DI^ and its seven focal dimerization residues in 159 species representing the taxonomic diversity of the order Mycobacteriales, the class Actinomycetes, and the phylum Actinomycetota. The seven residues critical for dimerization are highly conserved, not just within the order Mycobacteriales, but throughout the class Actinomycetes (Fig. 6A and B, and Supplementary Figs S12–S14). Most substitutions involve side chains of similar size and charge. Some residues show repeated evolution to the same side chain, while other, biochemically similar, side chains are never observed. Together, these observations suggest that the seven focal residues are under strong negative selection to maintain dimerization function throughout the class Actinomycetes and that the universe of workable substitutions is limited. Indeed, it is possible to reconstruct the likely ancestral state of the dimerization interface at the origin of the Actinomycetes for six of the seven residues. In contrast to the order Mycobacteriales and class Actinomycetes, the conservation and presence of the dimer is much less clear in the phylum Actinomycetota, where only a minority of focal residues are well conserved.

While widespread conservation of the DnaA^DI^ dimer in the Actinomycetes indicates its physiological importance, the specific biological role of the dimer was not directly examined in this study. However, previous work on the DnaA^DI^ from *S. lividans* provides key insight into the matter. Specifically, these previous studies indicated that the *S. lividans* DnaA^DI^ oligomerizes and that this interaction is important for proper orisome formation [15, 16]. Indeed, *S. lividans* DnaA lacking domain I formed large nucleoprotein aggregates in the presence of DNA, suggesting the domain plays a role in the choreography of origin-complex assembly [15, 16]. *S. lividans* DnaA^DI^ contacts, in conjunction with the presence of a notably long domain II (>200 residues), were proposed to link the distally located DnaA-box clusters that comprise the *Streptomyces oriC*. Similar organizational and long-range oligomerization roles may be adopted by the DnaA^DI^s of other Actinomycetes bacteria, specifically tailored to the distinctive *oriC* architecture of these organisms. Future biochemical and cell biological studies will be required to resolve the precise molecular details of *oriC* organization and its relationship to DnaA^DI^ across Actinomycetes and beyond.

Notably, our data indicate that self-association of the Actinobacterial DnaA^DI^s is relatively weak, demonstrating low- to mid-micromolar range K_D_. We propose that DnaA^DI^ dimerization supports the cooperative assembly of DnaA molecules at *oriC*, where high-affinity DnaA^DI^ self-associations would lead to over-initiation or improper orisome architecture, likely to devastating effect. In this way, the Actinomycetes DnaA^DI^ dimer could act as a fine-tuned, cooperative trigger, driving the assembly of the orisome. Making more plausible this argument of fine-tuning is the observation that, despite differences in quaternary arrangement and interface chemistries, the Actinobacterial DnaA^DI^s exhibit similarly weak self-association affinities. This suggests that the strength of DnaA^DI^ dimerization may constitute a meaningful evolutionary constraint that can be satisfied by multiple and disparate chemical mechanisms. Functional import notwithstanding the weak association of the DnaA^DI^s likely contributes to the historical difficulties encountered in their capture and structural characterization. This experimental inconvenience, however, likely reflects biological necessity. Indeed, it is sensible to expect that dynamic biological phenomena are underpinned by regulatory mechanisms of commensurate intricacy, permitting multilevel calibration. Among these, weak, yet cooperative, interactions are known to constitute important nonlinear biological control operations [83], similar to what has been proposed here.

While several previous studies report that weak DnaA^DI^ self-interactions support cooperative assembly of DnaA at the origin, the mechanistic basis for this effect is incompletely understood. Indeed, it is not immediately obvious how a head-to-head, formally monovalent interaction, like that exhibited by the DnaA^DI^ dimer, could give rise to cooperativity. One speculative solution to this dilemma invokes network-driven emergent cooperativity. In this view, the accumulation of DNA-bound DnaA increases the local concentration of the DnaA^DI^ in the neighborhood of *oriC*. Consequently, DnaA^DI^ dimer formation becomes increasingly probable. These domain I self-interactions suppress DnaA escape from the *oriC* region and, thus, promote *oriC* rebinding. Lending credence to this idea, a recent study demonstrated that fusion of a homodimerization domain to the arms of an IgG dramatically enhanced antibody binding to an antigen-labeled target surface [84]. In this way, a network of rapidly exchanging DnaA^DI^ dimer interactions might increase the effective concentration of DnaA in the vicinity of *oriC*, thereby increasing apparent DnaA-*oriC* association rates [85, 86]. Notably, by this model, DnaA^DI^ dimerization and *oriC* rebinding are mutually reinforcing and, thus, could give rise to cooperative-like phenomena. Additionally, this model immediately suggests a function for the understudied but much-discussed DnaA domain II length. Specifically, in this model, the domain II length would function as a mass action modulator, tuning the effective concentration of domain I and its spatially available binding partners, and, consequently, its monomer–dimer equilibrium [87, 88]. Indeed, it is tempting to speculate that the length of the domain II is under evolutionary pressure, varying non-randomly across bacterial taxa.

In addition to its role in orisome cooperative assembly, it is interesting to consider the implications of DnaA^DI^ dimerization on the interactions of the domain with its known binding partners. Motivating this contemplation, our HDX-MS data indicate that dimerization stabilizes DnaA^DI^ protomers. Specifically, in all tested DnaA^DI^ proteins, dimerization induced a significant decrease in deuterium exchange in ⍺3 (and, to a lesser degree, other secondary structure elements), consistent with the stabilization of the helix. Of note, the interaction of *E. coli* replicative helicase DnaB with the DnaA^DI^ has been mapped to DnaA residues E21 and F46 [29, 30]. These residues reside on helices ⍺2 and ⍺3 of the *E. coli* DnaA^DI^, corresponding to the same Actinomycetes DnaA^DI^ regions that our HDX-MS data indicate are stabilized upon dimerization. Indeed, the DnaA^DI^ ⍺2 and ⍺3 helices have been proposed to form a conserved protein–protein interaction surface [30]. Thus, dimerization may, by conformationally constraining these helices, stabilize and thereby promote interactions at this interface.

In summary, this study has uncovered a conserved mode of dimerization of the DnaA^DI^ across the class Actinomycetes. These results have important implications on our understanding of DnaA activity across Actinomycetes and serve as a platform on which subsequent cellular, biochemical, and structural studies can be built. In addition, these studies raise intriguing questions about the evolution of the DnaA^DI^; the relationship between the DnaA^DI^ and DnaA^DII^; and, more broadly, the evolutionary pressures on protein dimer interfaces.

## Supplementary Material

gkag596_Supplemental_Files

## Data Availability

Structure factor and coordinate files for all structures reported here have been deposited in the RCSB Protein Data Bank [89]; accession codes are available in Supplementary Table S2. The *C. bovis* DnaA^DI^ (S89C) MS data have been deposited to ProteomeXchange Consortium via the PRIDE partner repository with the dataset identifier PXD074582 [90, 91]. All HDX-MS data have been deposited to the ProteomeXchange Consortium via the PRIDE partner repository with the dataset identifier PXD074705. hdITC, SEC, and CD source data are available from the corresponding author upon reasonable request.
