# Supplementary material for: Structures of DnaA domain I reveal a dimer conserved across Actinomycetes
Source: Nucleic Acids Res. 2026 Jun 16;54(11):gkag596. doi: 10.1093/nar/gkag596 (PMC13270200; doi:10.1093/nar/gkag596)
Supplement: gkag596_Supplemental_Files [file gkag596_supplemental_files.zip › ELLIS-JUNE6-REVISE-SUPPLEMENTARY.pdf]

Structures of DnaA domain I reveal a dimer conserved across Actinomycetes

Porter K. Ellis[1], Bindu Y. Srinivasu[2], Jose Chavez Orozco Jr.[1], Gregory A. Wray[3], Thomas E. Wales[2], Maria A. Schumacher[1,*]

[1]Department of Biochemistry, 307 Research Dr., Box 3711, Duke University Medical Center, Durham, NC 27710, USA.

[2]Department of Chemistry and Chemical Biology, Northeastern University, Boston, MA 02115, USA.

[3]Department of Biology, 130 Science Dr., Duke University, Durham, NC 27708, USA.

* To whom correspondence should be addressed. Email: maria.schumacher@duke.edu

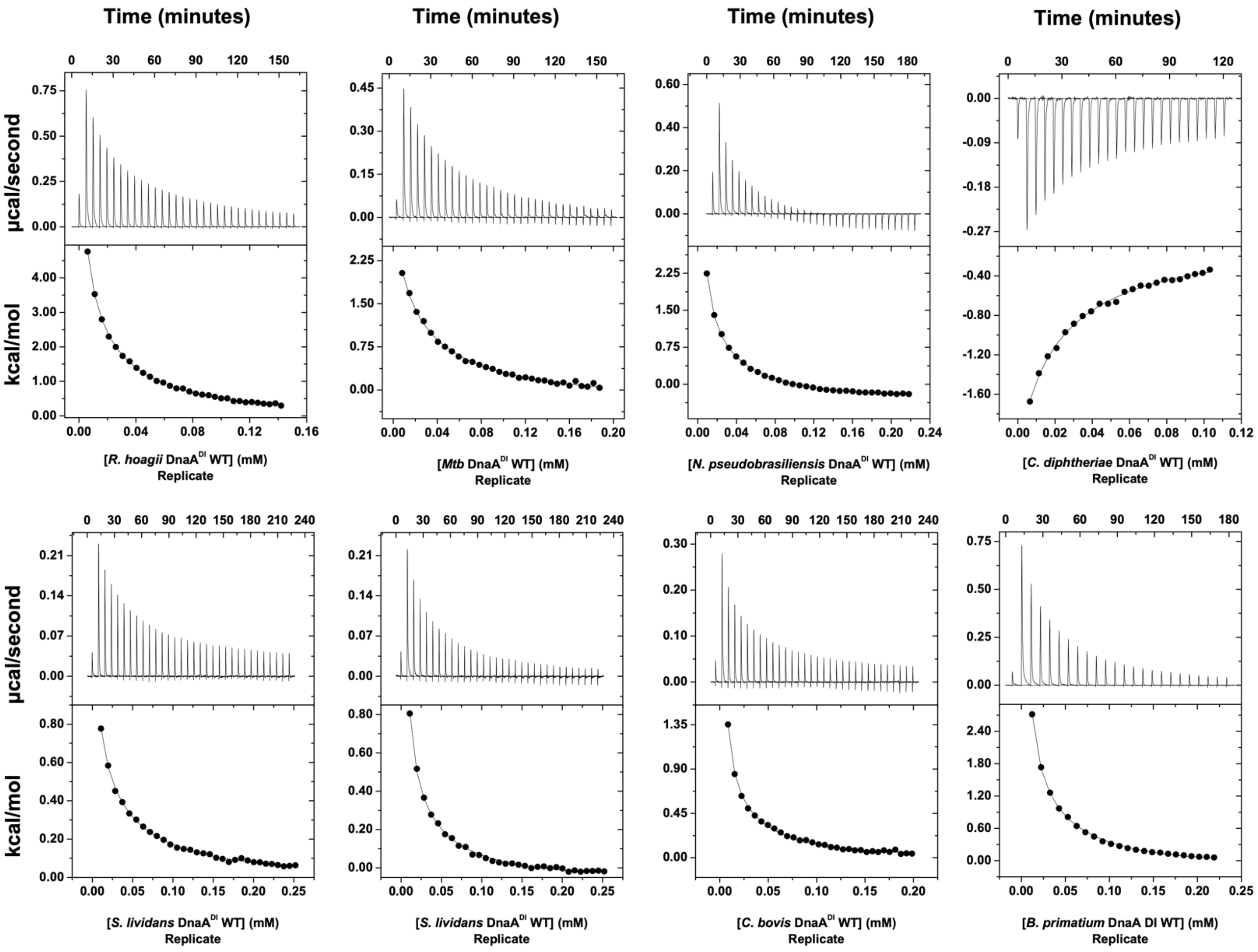

Figure S1. Actinomycetes DnaA^{DI} hdITC experiment replicates. Replicate hdITC experiments for Actinomycetes DnaA^{DI} species examined in Figs. 2 and 3 of this study.

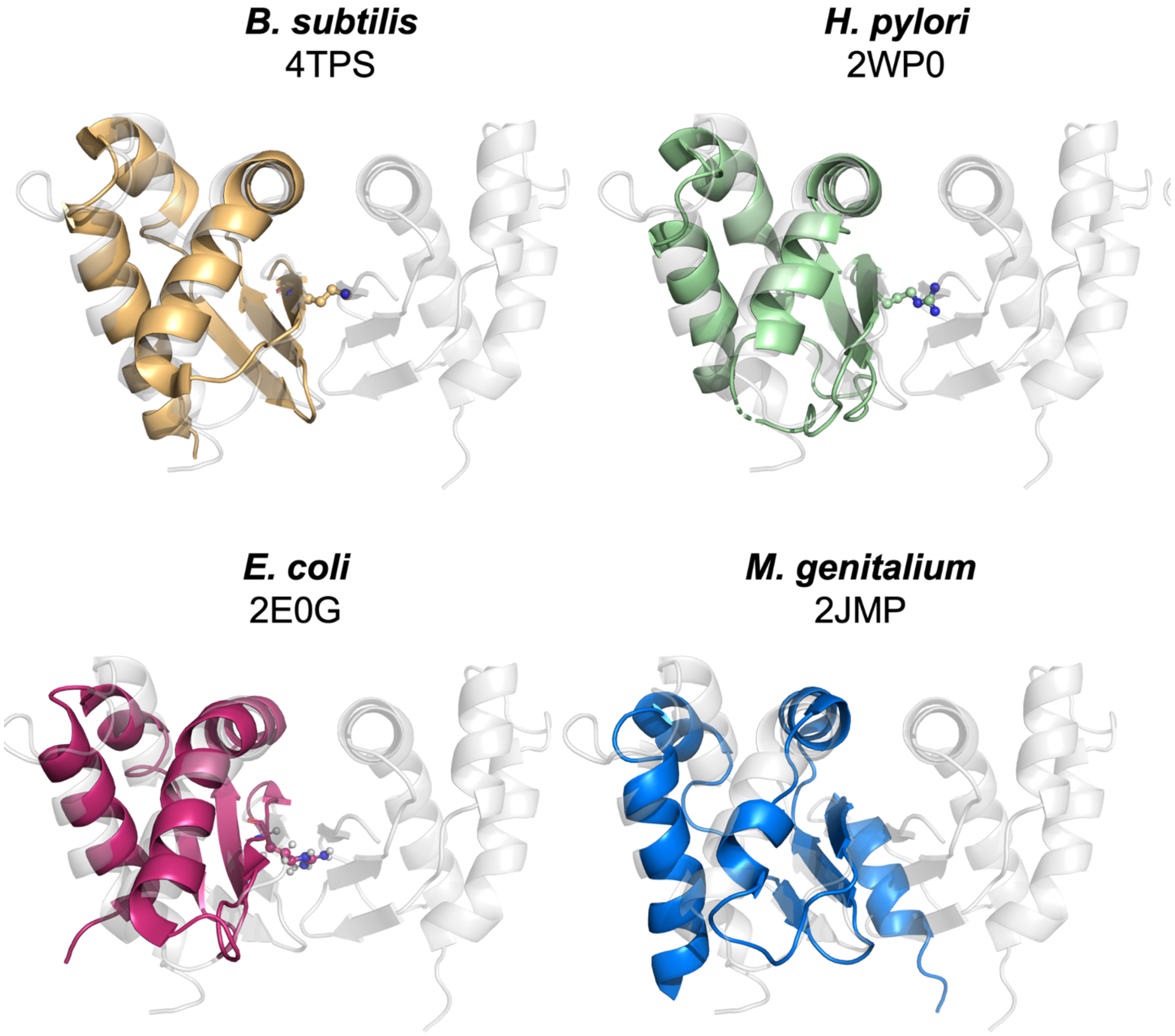

Figure S2. Comparison of solved DnaA^{DI} structures to the *Rh* DnaA^{DI}. The known structures (PDB entries noted in the figure) of four DnaA^{DI} proteins were aligned to a single protomer of the *Rh* DnaA^{DI} dimer by secondary structure matching as implemented in PDBeFold. For the *B. subtilis, H. pylori,* and *E. coli* DnaA^{DI}s, residues incompatible with the Actinomycetes dimerization mode are shown in ball-and-stick representation. The C-terminal helix of the *M. genitalium* DnaA^{DI} similarly occludes the Actinomycetes DnaA^{DI} dimerization interface.

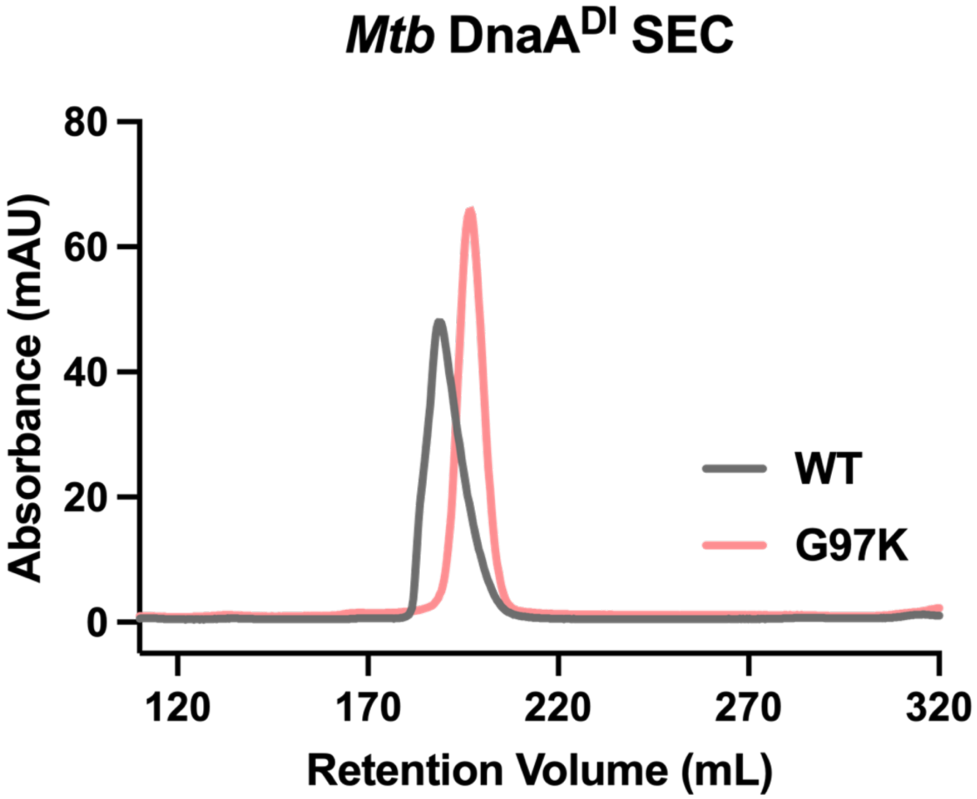

Figure S3. The *Mtb* DnaA^{DI} forms a dimer in solution. Size exclusion chromatography (SEC) analyses of WT and G97K *Mtb* DnaA^{DI} proteins (0.75 mg/mL). Proteins were eluted from the column at 1 mL/min. Consistent with the *R. hoagii* DnaA^{DI} structural dimer, the *Mtb* DnaA^{DI} WT species exhibits a decreased retention time on the SEC column relative to its G97K counterpart, suggesting the *Mtb* DnaA^{DI} forms a multimer.

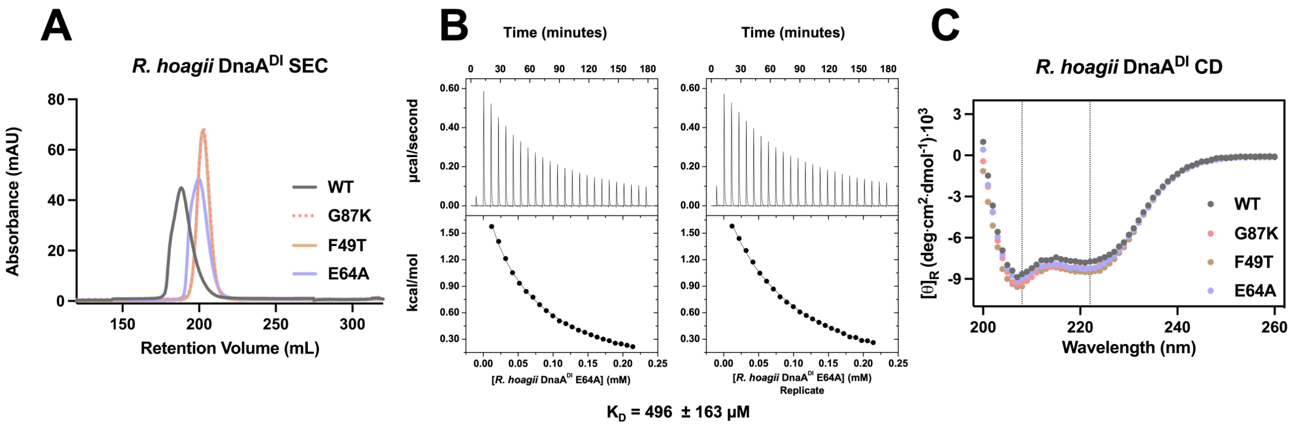

Figure S4. Mutations to the *Rh* DnaA^{DI} dimerization elements impair self-interactions. (A) Size exclusion chromatography (SEC) analyses of WT, G87K, F49T, and E64A *Rh* DnaA^{DI} proteins (0.75 mg/mL). Samples were eluted at two mL/min. **(B)** hdITC analysis of the *Rh* DnaA^{DI} E64A mutant protein. **(C)** CD spectra of the *Rh* DnaA^{DI} WT, G87K, F49T, and E64A proteins at 17.5 µM.

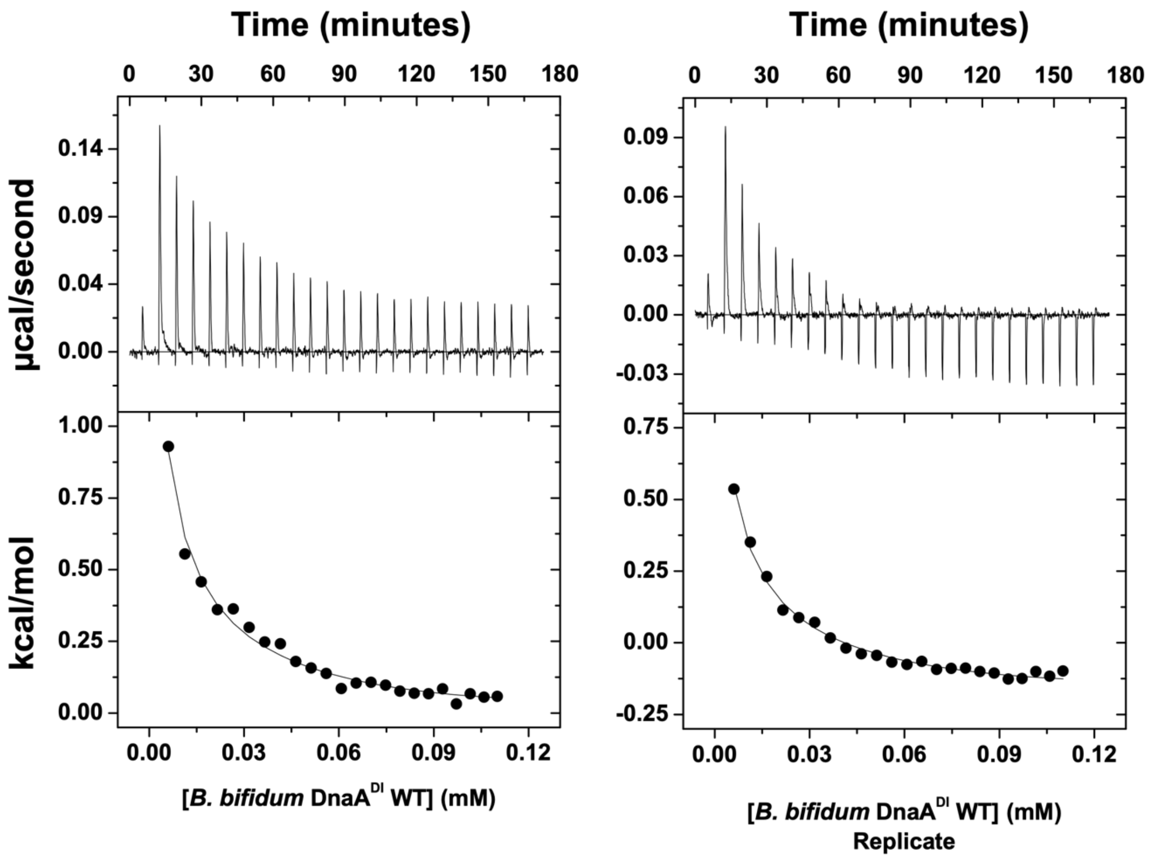

Figure S5. hdITC analysis of the *B. bifidum* DnaA^{DI} is consistent with dimer formation. Representative homodimer-dissociation isothermal titration calorimetry (hdITC) thermograms of the WT *B. bifidum* DnaA^{DI} (which encodes alanine at the steric selection site). The dissociation constant is given by K_D = 33 ± 4 µM. The reported dissociation constant is the average of two replicate experiments with error given by the standard deviation of the measurements. Note, under the assay conditions, the *B. bifidum* DnaA^{DI} formed microcrystals. Thus, data analysis is complicated by potential enthalpic contributions from isodesmic (indefinite) dissociation and/or crystalline dissolution. Accordingly, dimer-dissociation model fits for this experiment should be interpreted with some caution.

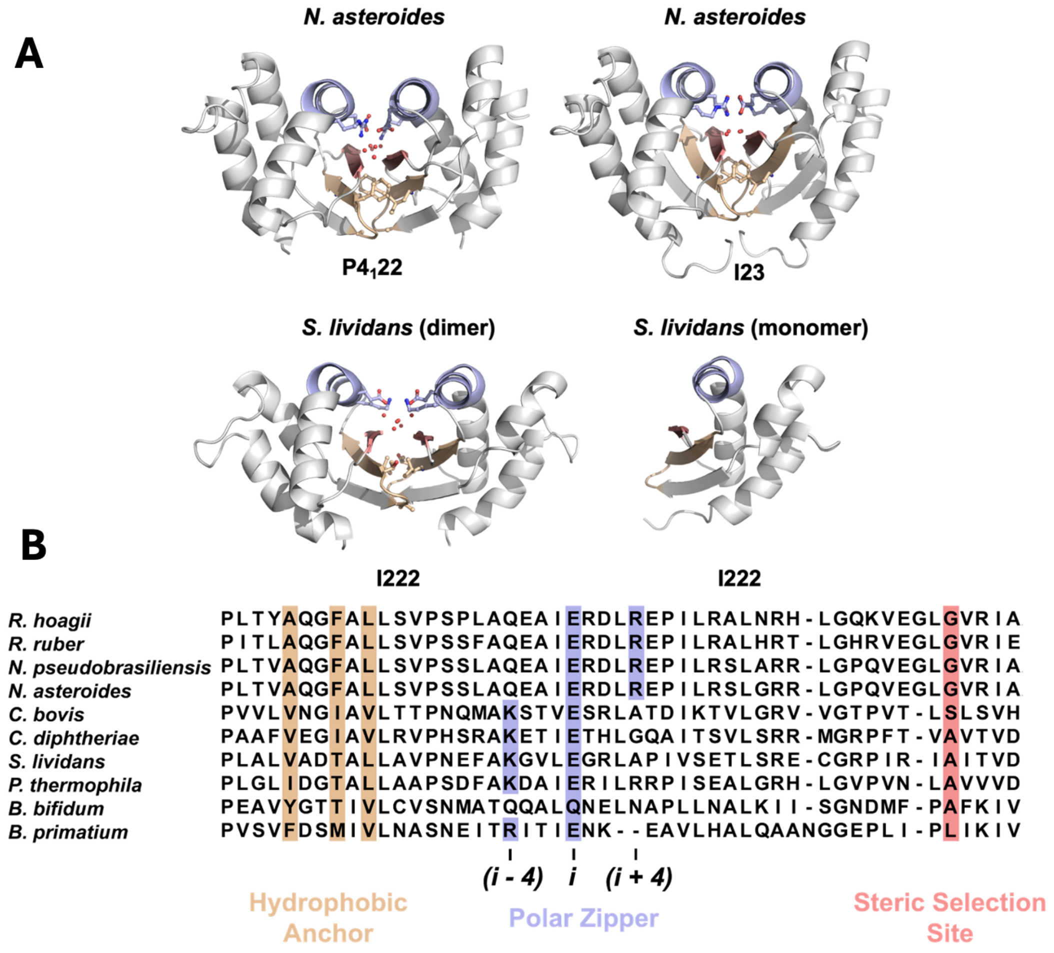

Figure S6. Polymorph structures of the *N. asteroides* and *S. lividans* DnaA^{DI}. (A) The structures of the *N. asteroides* and *S. lividans* DnaA^{DI}s were solved in multiple crystal forms. The same general dimer interface and supporting dimerization elements are found in these distinct polymorphs. The polar zipper, intermolecular-sheet, and hydrophobic anchor are shown in light blue, salmon, and wheat, respectively. The I222 *S. lividans* DnaA^{DI} structure, in addition to a dimer, captured a monomer of the protein. The overall structure of the monomer is similar to its dimeric counterpart, though, with elevated B-factors, possibly suggesting the structure is stabilised upon dimerization. **(B)** Multiple-sequence alignment reproduced from Figure 4B of the main text. A multiple-sequence alignment, generated with the ClustalOmega webserver, of the DnaA^{DI} proteins for which structures are reported in this study.

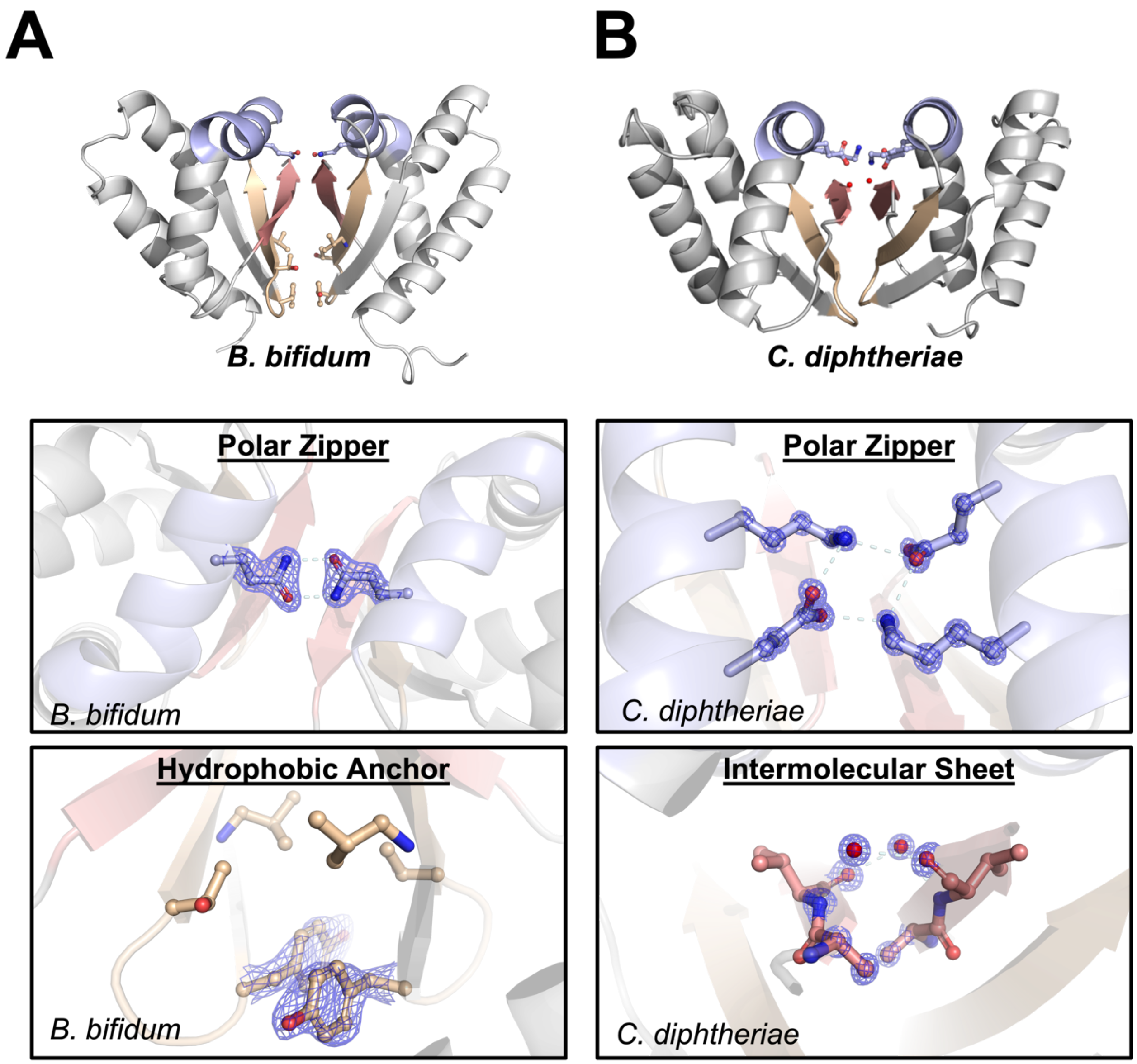

Figure S7. Subtle differences in dimerization element chemistries have evolved to support the Actinomycetes DnaA^{DI} dimer. (A) The *B. bifidum* DnaA^{DI} dimer is supported by glutamine-glutamine side chain bidentate hydrogen bonding, differing from the salt-bridge pattern exhibited in other polar zippers described in this study. Further, the *B. bifidum* DnaA^{DI} hydrophobic anchor is notable for its tyrosine-tyrosine pi-stacking interaction and amphipathic threonine that resides at the solvent-dimer interface boundary. The threonine methyl moiety is directed inward toward the hydrophobic anchor while its hydroxyl group is directed outward toward the solvent. Residues of interest are shown in ball-and-stick representation with $2mF_O - DF_C$ electron density (blue mesh) contoured at 1.5 σ with a 1.6 Å carve radius, included for select regions. **(B)** The *C. diphtheriae* DnaA^{DI} dimer polar zipper motif consists of an ionic cluster comprised of two lysine and two glutamate residues; the steric selection site of the *C. diphtheriae* DnaA^{DI} domain is occupied by alanine. $2mF_O - DF_C$ electron density of the sub-Ångstrom resolution structure is shown in blue mesh and contoured at 3.0 σ with a 1.0 Å carve radius for the intermolecular sheet region and 1.2 Å carve radius for the polar zipper region.

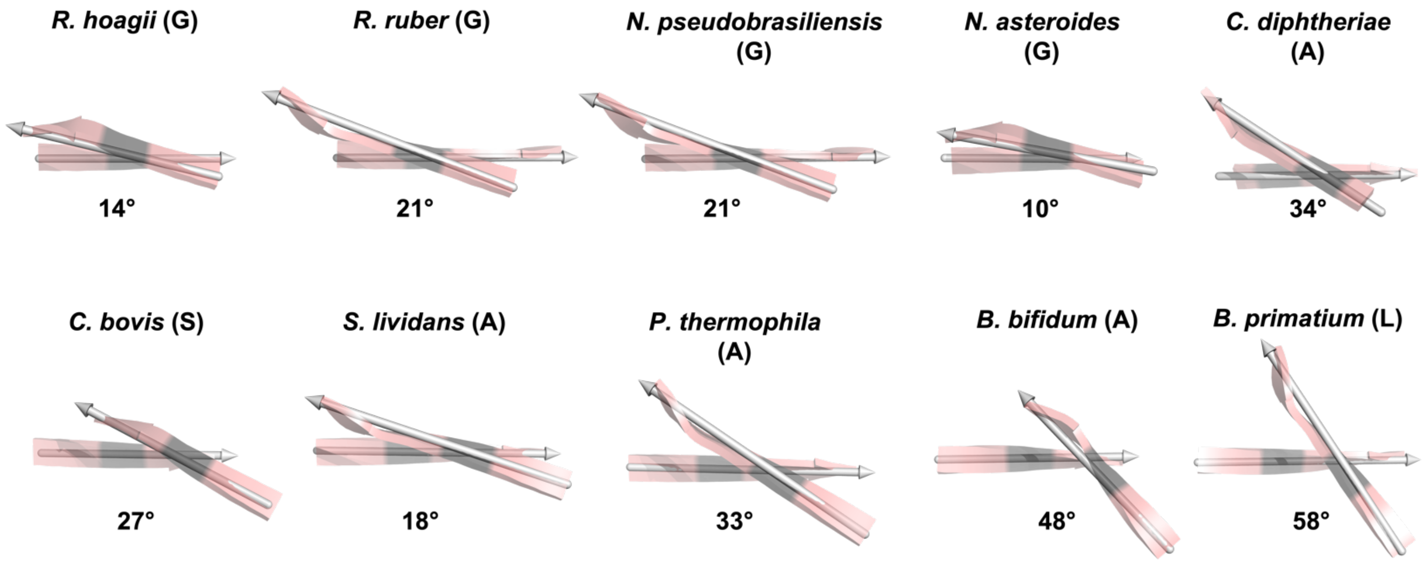

Figure S8. Differences in quaternary arrangement support accommodation of non-glycyl residues at the steric selection site of Actinomycetes DnaA^{DI} dimers. The Actinomycetes DnaA^{DI} dimer β3-β3′ interaction register and angle were analysed. For each dimer, the β3 and β3′ elements are shown in salmon and the steric selection site position is indicated in gray. Dimers encoding glycine at the steric selection site exhibit a face-to-face configuration of selection site residues. In contrast, dimers encoding non-glycyl residues exhibit lateral displacement of the selection site position. The orientation of the β3 and β3′ elements were computed using the AngleBetweenHelices program (Materials and Methods) and visualized as white vectors. The β3-β3′ interaction angle, defined as the angular offset from 180°, was computed using the same program.

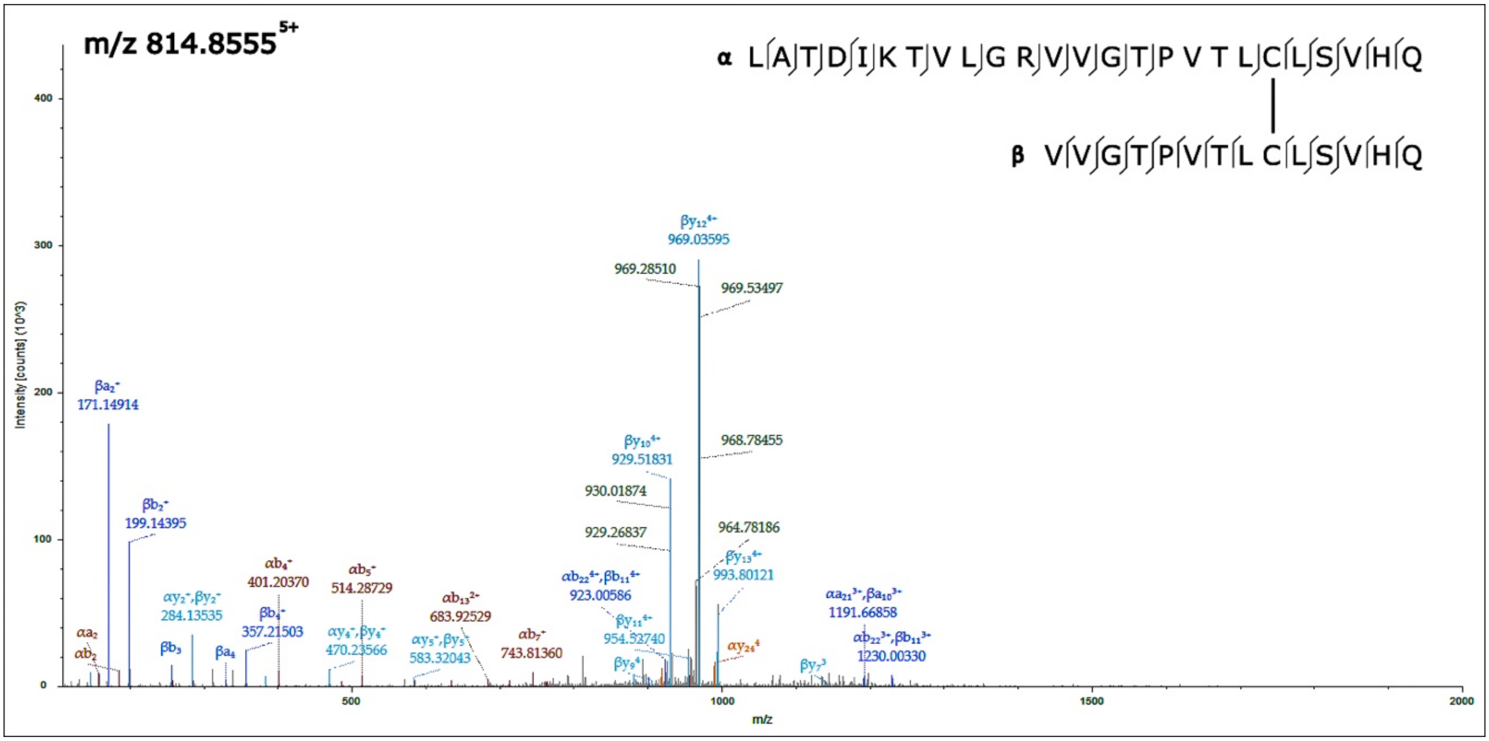

Figure S9. Tandem mass spectrometry confirms the *C. bovis* DnaA^{DI} dimer disulfide linkage. MS/MS spectrum of the disulfide-linked *C. bovis* DnaA^{DI} (S89C) tryptic fragment observed at m/z = 814.8555 $^{5+}$. The corresponding structure of the precursor ion is shown in the upper right. Identified MS/MS product ion series are annotated in the spectrum (α = LATDIKTVLGRVVGTPVTLCLSVHQ; β = VVGTPVTLCLSVHQ) and the corresponding fragmentation pattern is annotated on the precursor structure.

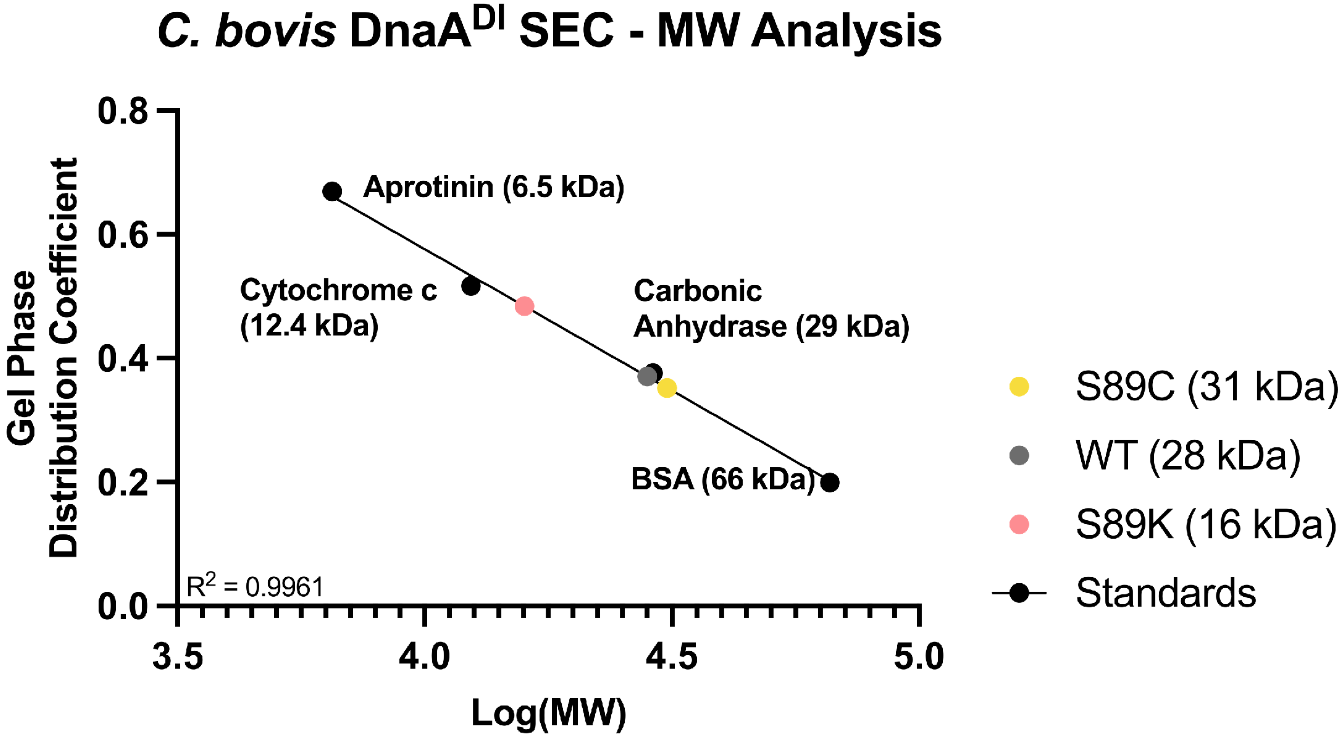

Figure S10. Gel phase distribution analysis reveals the *C. bovis* DnaA^{DI} forms a dimer. Approximate molecular weights (MW) of *C. bovis* DnaA^{DI} WT, S89K, and S89C species (at 2.0 mg/mL) were determined using SEC gel phase distribution analysis. The S89K and S89C mutants ran with apparent MWs of 16 and 31 kDa, respectively, consistent with monomer and dimer species. The WT species migrated with an apparent MW of 28 kDa, similar to the S89C protein, suggesting the WT species also forms a dimer. Gel filtration column calibration was performed by running protein standards including aprotinin, cytochrome c, carbonic anhydrase, and bovine serum albumin. Standards and samples were eluted at 2 mL/min.

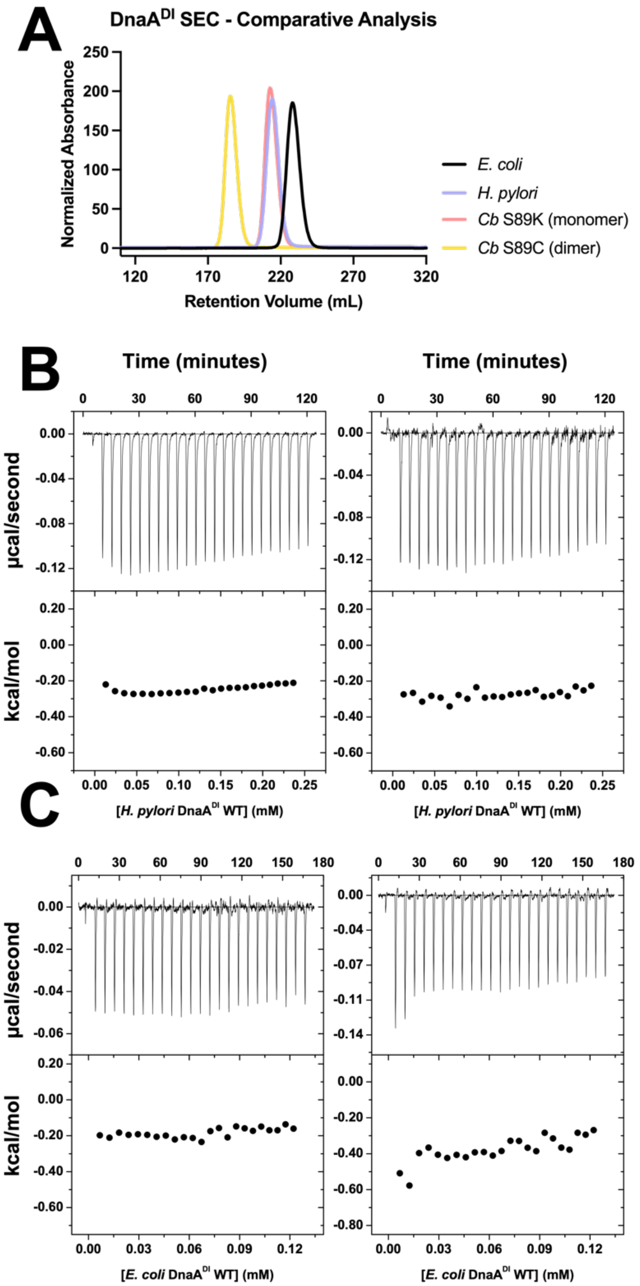

Figure S11. Size exclusion chromatography and homodimer-dissociation ITC (hdITC) experiments suggest *H. pylori and E. coli* DnaA^{DI} do not form dimers under the analysed conditions. (A) SEC analysis was performed on the *H. pylori* and *E. coli* DnaA^{DI}s at 2.0 mg/mL. Samples were eluted at two mL/min. Retention volumes were compared to those of *C. bovis* DnaA^{DI} (S89C) (which forms a dimer) and S89K (which forms a monomer) species run under the similar conditions. The *H. pylori* and *E. coli* proteins demonstrated similar or increased column retention times, respectively, to the *C. bovis* DnaA^{DI} S89K, suggesting these species form monomers under these conditions. **(B)** hdITC experiments performed on the *H. pylori* DnaA^{DI} at ~1.4 mM do not reveal dilution-associated heats of dimer-dissociation, suggesting this species does not form dimers under the examined conditions. **(C)** hdITC experiments performed on *E. coli* DnaA^{DI} at 700 μM do not reveal dilution-associated heats of dimer-dissociation, suggesting this species does not form dimers under the examined conditions.

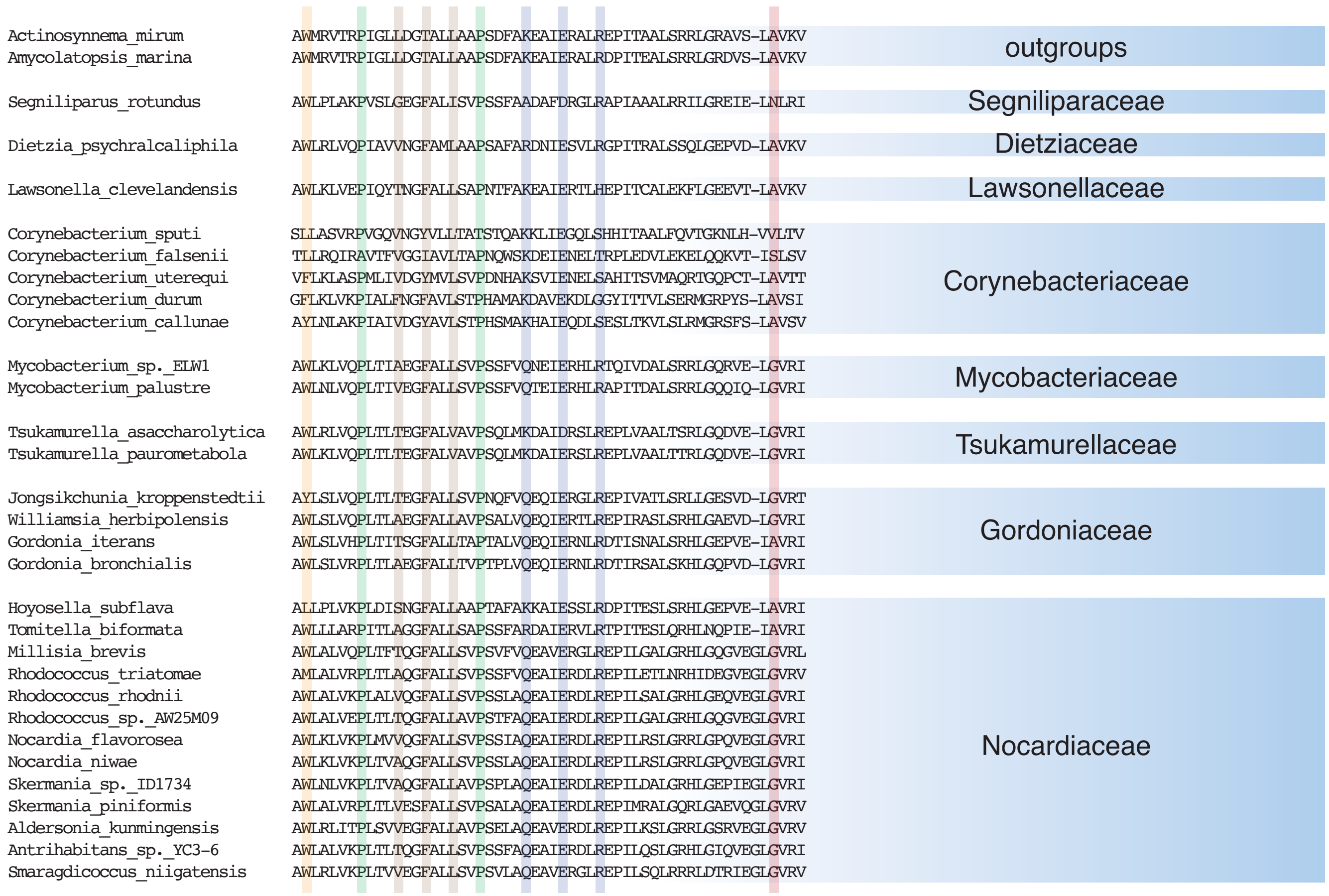

Figure S12. Alignment within the order Mycobacteriales. This alignment shows the central part of domain I of DnaA from 29 accessions representing broad taxonomic diversity within the order Mycobacteriales, along with 4 accessions from the class Acidimicrobia as an outgroup (Supplementary Table 4). Residues of interest are highlighted in colors consistent with main text Figure 6.

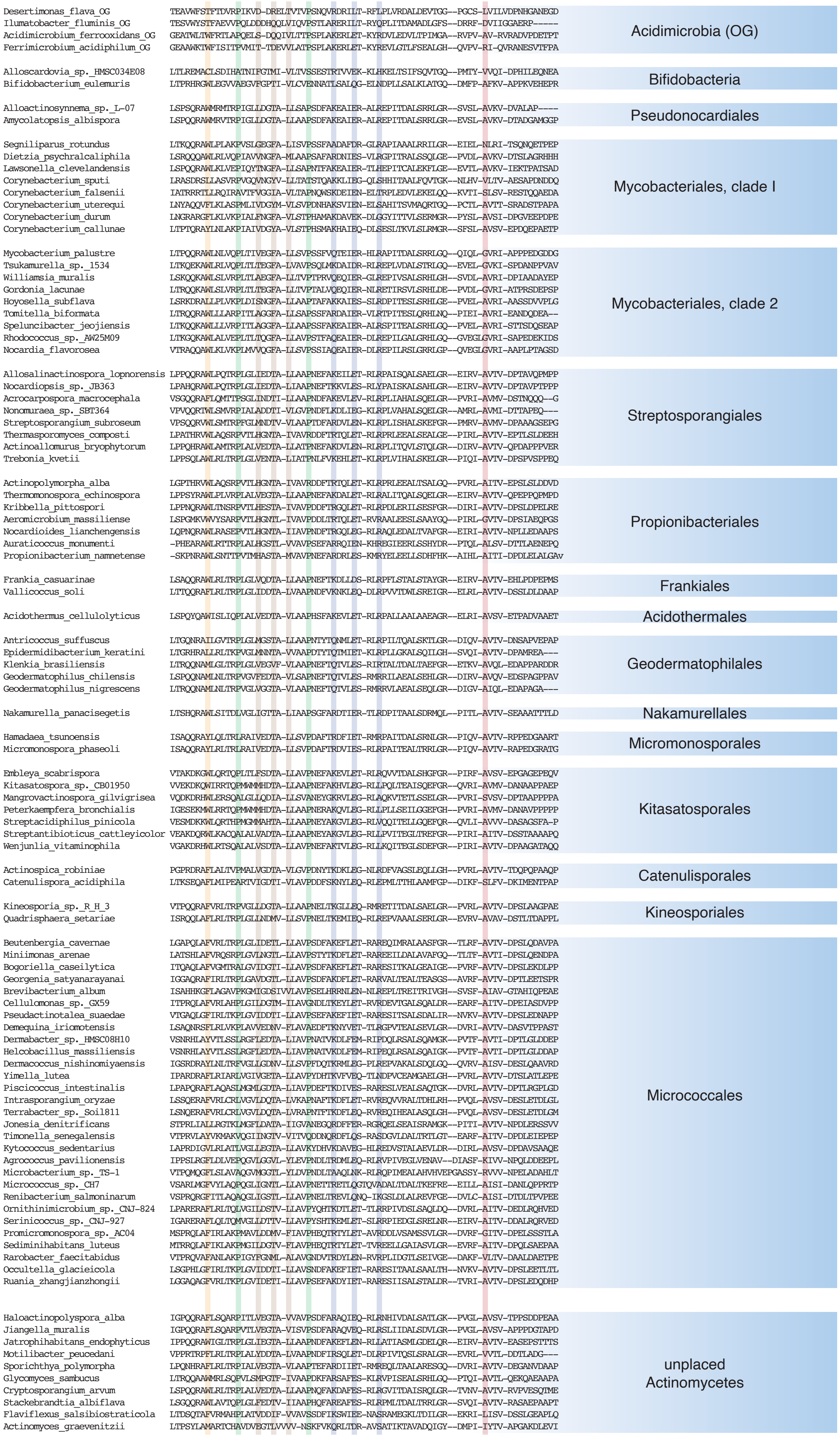
Desertimonas_flava_OG
Ilumatobacter_fluminis_OG
Acidimicrobium_ferrooxidans_OG
Ferrimicrobium_acidiphilum_OG
Acidimicrobia (OG)
Alloscardovia_sp._HMSC034E08
Bifidobacterium_eulemuris
Bifidobacteria
Alloactinosynnema_sp._L-07
Amycolatopsis_albispora
Pseudonocardiales
Segniliparus_rotundus
Dietzia_psychralcaliphila
Lawsonella_clevelandensis
Corynebacterium_sputi
Corynebacterium_falsenii
Corynebacterium_uterequi
Corynebacterium_durum
Corynebacterium_callunae
Mycobacteriales, clade I
Mycobacterium_palustre
Tsukamurella_sp._1534
Williamsia_muralis
Gordonia_lacunae
Hoyosella_subflava
Tomitella_biformata
Speluncibacter_jeojiensis
Rhodococcus_sp._AW25M09
Nocardia_flavorosea
Mycobacteriales, clade 2
Allosalinactinospora_lopnorensis
Nocardiopsis_sp._JB363
Acrocarpospora_macrocephala
Nonomuraea_sp._SBT364
Streptosporangium_subroseum
Thermasporomyces_composti
Actinoallomurus_bryophytorum
Trebonia_kvetii
Streptosporangiales
Actinopolymorpha_alba
Thermomonospora_echinospora
Kribbella_pittospori
Aeromicrobium_massiliense
Nocardioides_lianchengensis
Auraticoccus_monumenti
Propionibacterium_namnetense
Propionibacteriales
Frankia_casuarinae
Vallicoccus_soli
Frankiales
Acidothermus_cellulolyticus
Acidothermales
Antricoccus_suffuscus
Epidermidibacterium_keratini
Klenkia_brasiliensis
Geodermatophilus_chilensis
Geodermatophilus_nigrescens
Geodermatophilales
Nakamurella_panacisegetis
Nakamurellales
Hamadaea_tsunoensis
Micromonospora_phaseoli
Micromonosporales
Embleya_scabrispora
Kitasatospora_sp._CB01950
Mangrovactinospora_gilvigrisea
Peterkaempfera_bronchialis
Streptacidiphilus_pinicola
Streptantibioticus_cattleyicolor
Wenjunlia_vitaminophila
Kitasatosporales
Actinospica_robiniae
Catenulispora_acidiphila
Catenulisporales
Kineosporia_sp._R_H_3
Quadrisphaera_setariae
Kineosporiales
Beutenbergia_cavernae
Miniimonas_arenae
Bogoriella_caseilytica
Georgenia_satyanarayanai
Brevibacterium_album
Cellulomonas_sp._GX59
Pseudactinotalea_suaedae
Demequina_iriomotensis
Dermabacter_sp._HMSC08H10
Helcobacillus_massiliensis
Dermacoccus_nishinomiyaensis
Yimella_lutea
Piscicoccus_intestinalis
Intrasporangium_oryzae
Terrabacter_sp._Soil811
Jonesia_denitrificans
Timonella_senegalensis
Kytococcus_sedentarius
Agrococcus_pavilionensis
Microbacterium_sp._TS-1
Micrococcus_sp._CH7
Renibacterium_salmoninarum
Ornithinimicrobium_sp._CNJ-824
Serinicoccus_sp._CNJ-927
Promicromonospora_sp._AC04
Sediminihabitans_luteus
Rarobacter_faecitabidus
Occultella_glacieicola
Ruania_zhangjianzhongii
Micrococcales
Haloactinopolyspora_alba
Jiangella_muralis
Jatrophihabitans_endophyticus
Motilibacter_peucedani
Sporichthya_polymorpha
Glycomyces_sambucus
Cryptosporangium_arvum
Stackebrandtia_albiflava
Flaviflexus_salsibiostraticola
Actinomyces_graevenitzii
unplaced
Actinomycetes

Figure S13. Alignment within the class Actinomycetes. This alignment shows the central part of domain 1 of DnaA from 97 accessions representing broad taxonomic diversity within the class Actinomycetes, along with 2 accessions from other orders as outgroups (Supplementary Table 4). Residues of interest are highlighted in colors consistent with main text Fig. 6.

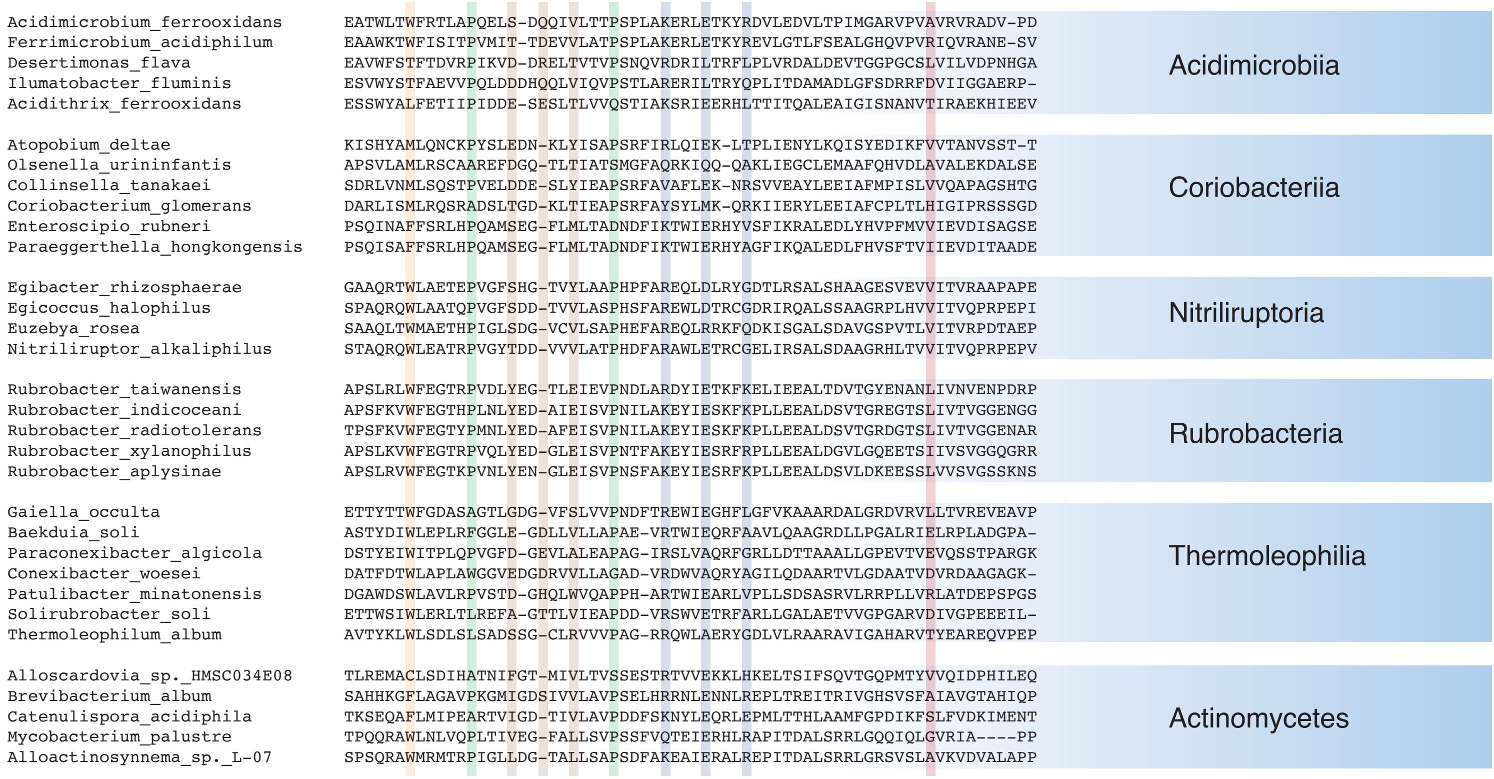

Figure S14. Alignment within the phylum Actinomycetota. This alignment shows the central part of domain I of DnaA from 32 accessions representing broad taxonomic diversity within the phylum Actinomycetota (Supplementary Table 4). Residues of interest are highlighted in colors consistent with main text Fig. 6.

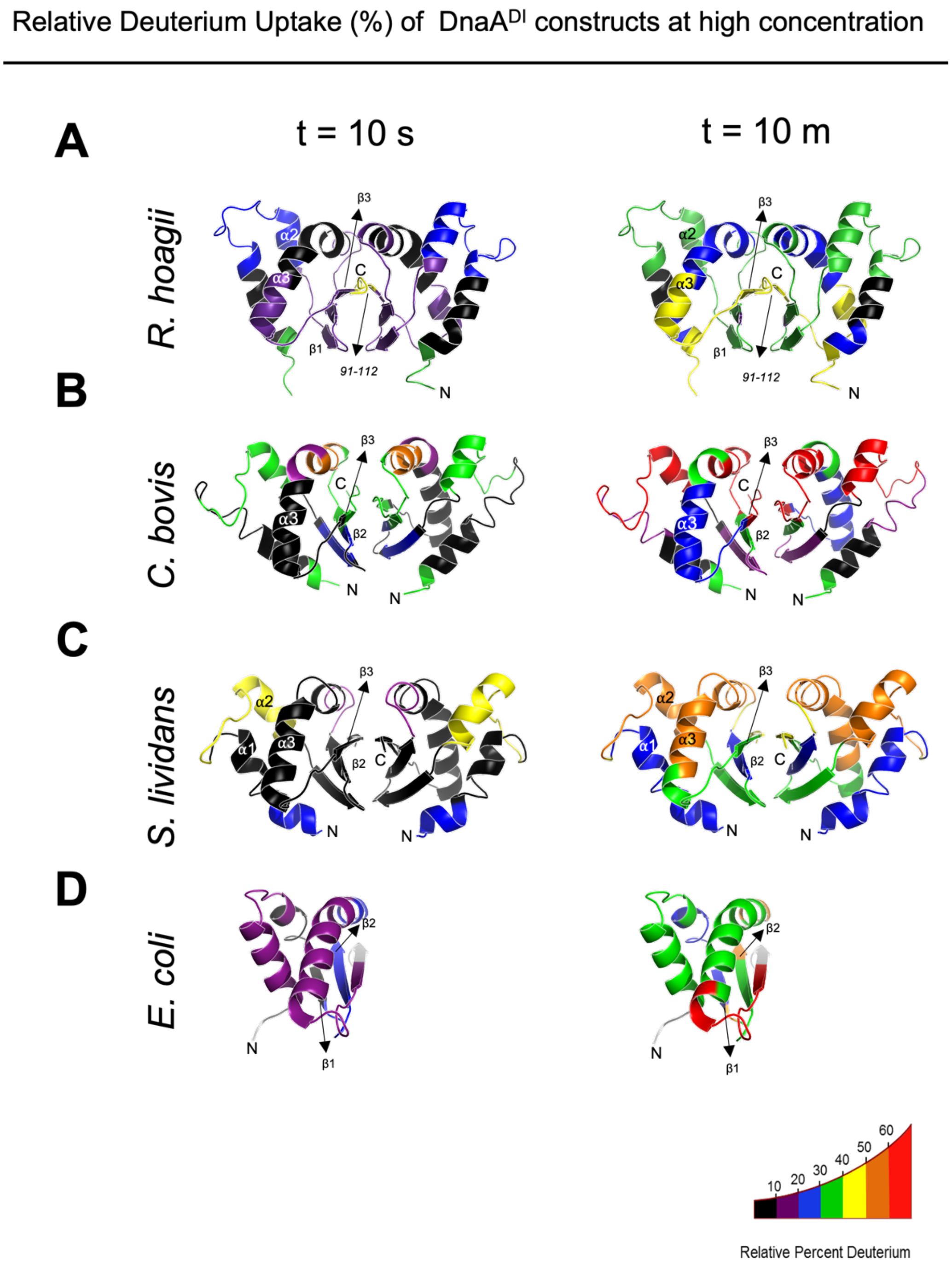

Figure S15. HDX-MS reveals intrinsically stable regions of DnaA^{DI} constructs. (**A-D**) Relative percent deuterium (RPD) values at 10 second- and 10-minute labelling time points are presented for select DnaA^{DI} constructs analysed under high-concentration conditions. RPD uptake data are mapped onto the corresponding structures and colored according to the scale shown. (**A**) *R. hoagii* DnaA^{DI} . (**B**) *C. bovis* DnaA^{DI}. (**C**) *S. lividans* DnaA^{DI}. (**D**) *E. coli* DnaA^{DI} (PDB ID 2E0G).

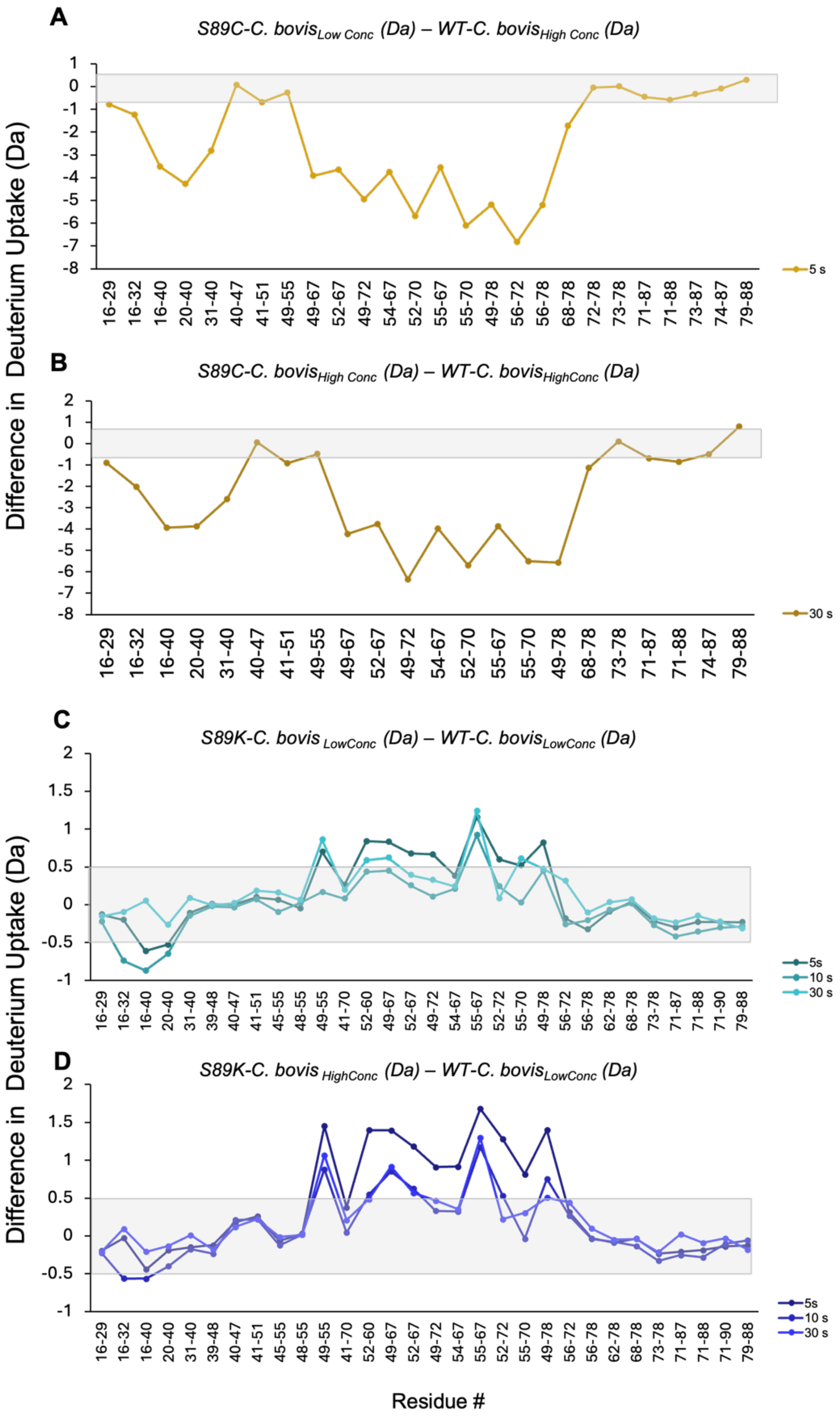

Figure S16. HDX-MS analysis of *C. bovis* DnaA^{DI} mutants with altered oligomeric states. **(A-D)** Deuterium difference plots showing the relative deuterium incorporation of *C. bovis* DnaA^{DI} constructs. **(A)** S89C *C. bovis*, 20 µM minus WT *C. bovis* 830 µM. **(B)** S89C *C. bovis*, 800 µM minus WT *C. bovis* 830 µM. (**C**) S89K *C. bovis*, 20 µM minus WT *C. bovis* 20 µM. **(D)** S89K *C. bovis*, 830 µM minus WT *C. bovis* 20 µM. The HDX MS data used to create this figure can be found in Supplementary Table 7.

Supplementary Table 1 (plasmid information) is provided as a *.xlsx file.

Supplementary Table 2 (protein crystal formation information) is provided as a *.xlsx file.

Supplementary Table 3 (merging and refinement statistics) is provided as a *.xlsx file.

Supplementary Table 4 (DnaA sequences/accessions) is provided as a *.xlsx file.

Table S5: Dissociation constants and Interface Surface Areas of the Actinomycetes DnaA^{DI}s

Organism of origin	Steric Selection Site	Interface Surface Area (ISA, Å^2)	Dissociation Constant (K_D, μM)
R. hoagii	G	617	45 ± 3
R. ruber	G	715	-
N. asteroides	G	649	-
N. pseudobrasiliensis	G	650	28 ± 6
C. diphtheriae	A	530	117 ± 6
C. bovis	S	439	21 ± 1
S. lividans	A	464	52 ± 26
P. thermophila	A	477	-
B. bifidum	A	486	**33 ± 4**
B. primatium	L	519	49 ± 10

Interface surface area (ISA) calculations were carried out using the PDBePISA webserver accessed on 12 January 2026 with default settings. ISA calculations were performed on the hexagonal *S. lividans* and trigonal *N. asteroides* DnaA^{DI} polymorphs; ISA calculations were performed on the *B. primatium* DnaA^{DI} dimer formed by Chains B and D. Dissociation constants are from hdITC experiments reported in the main text. Error is given by the standard deviation of n=2 or n=3 independent measurements.

Per the PDBePISA webserver, ISA is defined as the "difference in total accessible surface areas of isolated and interfacing structures divided by two."

The reported K_D for the *B. bifidum* DnaA^{DI} dimer should be interpreted with caution (see Supplementary Fig. 5 for additional information).

Table S6. Residues present at positions of interest across three scales of divergence.

Position	Mycobacteriales		Actinomycetes		Actinomycetota	
	common	*alternatives*	*common*	*alternatives*	*common*	*alternatives*
dimerization						
hydrophobic core 1	V, A, T (0.90)	G, F, S	V, L (0.46)	M, F, I, H, A, G, T, Y, S	S, D (0.41)	T, E, Y, G, A, F, I, V, L
hydrophobic core 2	F (0.86)	Y, I	T, F (0.84)	M, Y, I, V, S, N, L	?	?
hydrophobic core 3	L (0.90)	I, V	L, V (0.82)	M, I, F, Y, A	V (0.38)	L, T, Y, M, E, S, A, W, R
polar zipper 1 (**i - 1**)	K ,Q (0.90)	A, R	K, R, Q (0.97)	L, A	R, K (0.75)	Q, V, Y
polar zipper 2 (i)	E (0.93)	D	E (0.92)	Q, D	E (0.69)	L, Q, M, D, R, A
polar zipper 3 (**i + 1**)	R (0.80)	H, S, T, G	R (0.74)	H, N, S, T, G, L, V, E, P, N, A	R (0.25)	L, Q, T, A, V, G, H, E
steric selection	A, G (0.90)	S, N	A, G (0.76)	V, S, L, I, K, R	none	A, R, L, D, T, V, H, I, E, S
tertiary structure?						
tryptophan	W (0.83)	L, F, Y	W (0.54)	C, L, F, Y, I, V	W (0.63)	T, L, M, F, C
proline 1	P (0.97)	A	P (0.73)	A, L, M	P (0.72)	A, F, W, L
proline 2	P (0.97)	T	P (0.87)	T, S, E, A, G, Q, K	P (0.81)	Q, S, D, G

This table lists every residue present in the alignment at each scale of divergence (Figs. S12-S14). Residues present at a frequency of 0.20 or higher are considered "common" and their collective frequency at that scale is shown in brackets; all other residues are "alternatives". Note that it was not possible to confidently align residues corresponding to the hydrophobic core 2 position across the Actinomycetota due to a combination of low sequence conservation and multiple adjacent indels (Fig. S14).

Supplementary Table 7 (HDX-MS) is provided as a *.xlsx file.

Supplemental Discussion: Phylum Actinomycetota DnaA^{DI} Evolution

In contrast to the order Mycobacteriales and class Actinomycetes, the conservations and presence of the dimer observed in this study is much less clear in the phylum Actinomycetota, where only a minority of focal residues are well conserved. Only two residues comprising the salt bridge are well conserved among classes, while the residues around the second and third positions of the hydrophobic core remain generally hydrophobic. Substitutions at the other focal positions often involve changes in size and charge properties. Importantly, this includes some large side chains at the steric selection site (e.g., Arg, His) that are likely incompatible with dimerization (though, some residues, e.g.: valine, are also present, which may be compatible with dimerization). Yet some other positions within domain 1, most notably a tryptophan and two prolines, remain very well conserved among classes, suggesting they play important role in stabilizing the DnaA^{DI} tertiary structure. Together, these observations indicate that dimerization may be possible in some species outside the Actinomycetes, but widespread conservation of dimerization among the classes of Actinomycetota seems unlikely. Clearly, direct evidence from crystal structures or functional assays will be required to resolve both the true phylogenetic extent of the dimerization interface.
